# Supplementary material for: Chinese herbal therapy in the management of rhinosinusitis—A systematic review and meta-analysis
Source: PLoS One. 2022 Dec 1;17(12):e0278492. doi: 10.1371/journal.pone.0278492 (PMC9714754; doi:10.1371/journal.pone.0278492)
Supplement: S1 Table — Including: Databases that were searched and PubMed search terms for CHM for RS; List of excluded studies with reasons; Ingredients of the CHM interventions, manufacture and dosage used in the included studies and funding; Main ingredients of the Chinese herbal medicines; Risk of bias judgements for included studies; SNOT: Meta-analysis results for chronic rhinosinusitis at end of treatment and changes within treatment and control groups; SNOT-20-subscales: Meta-analysis results for CRS at end of treatment change within treatment groups and control groups; VAS-TNS Meta-analysis results for CRS at end of treatment and change within treatment and control groups; VAS-IS Meta-analysis results for CRS at end of treatment and change within treatment groups and control groups; LM Meta-analysis results for CRS at end of treatment and change within treatment and control groups; LK Meta-analysis results for CRS at end of treatment and change within treatment and control groups; MTT Meta-analysis results for CRS at end of treatment and change within treatment and control groups; MTR Meta-analysis results for CRS at end of treatment and change within treatment and control groups; SF-36 Meta-analysis results for CRS at end of treatment and change within treatment groups and control groups; and Details of reported adverse events from included studies. (DOCX) [file pone.0278492.s002.docx]

**S1 Table. Additional data**

**for**

**Chinese herbal therapy in the management of rhinosinusitis - A systematic review and meta-analysis**

**List of Tables of Additional Data**

S1 Table. Databases that were searched and PubMed search terms for CHM for RS

S2 Table. List of excluded studies with reasons

S3 Table. Ingredients of the CHM interventions, manufacture and dosage used in the included studies and funding

S4 Table. Main ingredients of the Chinese herbal medicines

S5 Table. Risk of bias judgements for included studies

S6 Table. SNOT: Meta-analysis results for chronic rhinosinusitis at end of treatment

S7 Table. SNOT-20 subscales: Meta-analysis results for CRS at end of treatment change within treatment groups and control groups

S8 Table. VAS-TNS Meta-analysis results for CRS at end of treatment and change within treatment and control groups

S9 Table. VAS-IS Meta-analysis results for CRS at end of treatment and change within treatment groups and control groups

S10 Table. LM Meta-analysis results for CRS at end of treatment and change within treatment and control groups

S11 Table. LK Meta-analysis results for CRS at end of treatment and change within treatment and control groups

S12 Table. MTT Meta-analysis results for CRS at end of treatment and change within treatment and control groups

S13 Table. MTR Meta-analysis results for CRS at end of treatment and change within treatment and control groups

S14 Table. SF-36 Meta-analysis results for CRS at end of treatment and change within treatment groups and control groups

S15 Table. Details of reported adverse events from included studies

**S1 Table. Databases that were searched and PubMed search terms for CHM for RS**

| **Databases and other resources that were searched** |
| --- |
| Searches were conducted for clinical trials assessing the effects of CHMs in the management of RS. Databases included: PubMed, Cochrane Central Register of Controlled Trials, EMBASE, AMED, CINAHL, Chinese Biomedicine, China Network Knowledge Infrastructure (CNKI), Wanfang Database and Chinese Scientific Journals Database). Each database was searched from its inception date until August 9th 2022.  In addition, targeted searches of the following sources were conducted: Australia New Zealand Clinical Trial Registry (ANZCTR); Chinese Clinical Trial Registry (ChiCTR) 中国临床试验注册中心; EU Clinical Trials Register (EU-CTR); ClinicalTrials.gov; Web of Science; ProQuest Dissertations & Theses Global (aka ProQuest Central) (from their respective inception dates until August 12th 2022); and reference lists in retrieved papers. |
| **PubMed search blocks** |
| Search Terms [All Fields]  Group 1: Disorder  "sinusitis"[MeSH Terms] OR "sinusitis"[All Fields] OR rhinosinusitis [All Fields] OR rhino-sinusitis [All Fields] OR "nose"[MeSH Terms] OR "nose"[All Fields] OR "rhino"[All Fields] AND ("sinusitis"[MeSH Terms] OR "sinusitis"[All Fields]  Group 2: Intervention  Traditional Chinese Medicine OR Chinese Traditional Medicine OR Chinese Herbal Drugs OR Chinese Drugs, Plant OR Medicine, Traditional OR Ethnopharmacology OR Ethnomedicine OR Ethnobotany OR Medicine, Kampo OR Kanpo OR TCM OR OR Medicine, Ayurvedic OR Phytotherapy OR Herbology OR Plants, Medicinal OR Plant Preparation OR Plant Extract OR Plants, Medicine OR Materia Medica OR Single Prescription OR Herbs OR Chinese Medicine Herb OR Herbal Medicine;  Group 3: Study type  “randomized controlled trial”[pt] OR “controlled clinical trial”[pt] OR “randomized”[tiab] OR “placebo”[tiab] OR “drug therapy”[sh] OR “randomly”[tiab] OR “trial”[tiab] OR “groups”[tiab]  Combined: Group 1 AND Group 2 AND Group 3 |

Abbreviations: RS: rhinosinusitis; CHM: Chinese herbal medicine.

**S2 Table. List of excluded studies with reasons**

| **Authors** | **Year** | **Title** | **Main reasons for exclusion** |
| --- | --- | --- | --- |
| Xu, K; Liang, C; Qi, X; et al | 2005 | A clinical observation on the effect of irrigating solution of Shuang Huang Lian on ESS for chronic sinusitis | not RCT |
| Liu, FX; Ye, HX; Wang, LL; Lin, J | 2016 | Effect of Sinusitis Mixture on Mucosa Cells after Functional Endoscopic Sinus Surgery | not RCT |
| Xiong, ZJ; Zhang, SQ; Fang, R | 2005 | Effect on the recovery process of nasal sinus mucosa with nasosinusitis and nasal polyps influenced by applying Chinese herbal preparation | not RCT |
| Wu, WQ; Huang, SG; Ma, Z; Zhang, YQ; Shi, JB | 2005 | Effects of irrigating solution of Sihuang on morphology and function of nasal mucosa following surgery for chronic sinusitis and nasal polyps | not RCT |
| Goos, KH; Albrecht, U; Schneider, B | 2006 | Efficacy and safety profile of an herbal drug containing nasturtium herb and horseradish root in acute sinusitis, acute bronchitis and acute urinary tract infection in comparison with other treatments in the daily practice/results of a prospective cohort study | not RCT, not CHM |
| Li, C; Zhao, Y; Liang, C; An, H | 2001 | Observations of the curative effect with various liquid for post operative irrigation of ESS of treating chronic sinusitis and nasal polyps | not RCT |
| Yen, HR; Sun, MF; Lin, CL; Sung, FC; Wang, CC; Liang, KL | 2015 | Adjunctive traditional Chinese medicine therapy for patients with chronic rhinosinusitis: a population-based study | not RCT |
| Sakurada, T; Ikeda, K; Takasaka, T | 1992 | Clinical effectiveness of Kampo medicine for chronic rhinitis and sinusitis (clinical observation) | not RCT |
| Cai, PP; Zhang, XY; Mou, LM | 2009 | Clinical observation of Jiawei Qianjin Weijing Tang granule in treating acute sinusitis | not RCT |
| Zhang, HJ; Li, SL | 2000 | Clinical observation of sinusitis mixture for treating sinusitis in 87 Cases | not RCT |
| Qian, JH; Chen, HE; Chen, GX; Yuan, Q; Bao, WW | 2001 | Clinical research of Bi Yuan San for treating sinusitis in children | not RCT |
| Wu, ZQ; Tian, J; Ma, BY; Wang, WZ; Li, XM | 1989 | Clinical research of Xiang Ju Tablet in treating sinusitis | not RCT |
| Liu, FX; Ye, HX; Wang, LL; Lin, J | 2016 | Effect of Sinusitis Mixture on Mucosa Cells after Functional Endoscopic Sinus Surgery | not RCT |
| Xiong, ZJ; Zhang, SQ; Fang, R | 2005 | Effect on the recovery process of nasal sinus mucosa with nasosinusitis and nasal polyps influenced by applying Chinese herbal preparation | not RCT |
| Huang, C; Miao, G; Wu, Z; Liu, J; Yu, G | 1996 | External use of bi yuan ning in 200 cases of nasosinusitis and rhinitis | not RCT |
| Gao, SJ; Du, JY | 1993 | Integrative medicine therapy for chronic maxillary sinusitis in 60 cases | not RCT |
| Son, MJ; Kwon, O; Kim, S; et al | 2018 | Safety and efficacy of Galgeun-tang-ga-cheongung-sinyi, a herbal formula, for the treatment of chronic rhinosinusitis: A study protocol for a randomized controlled trial | not RCT |
| Peng, Q; Qin, G; Hou, T; Liang, Z; Zhou, W | 2015 | Short-term efficacy observation on Chinese traditional medicine used after functional endoscopic sinus surgery for chronic sinusitis | not RCT |
| Peng, X; Zheng, S | 1992 | Sinusitis mixture combined with ultrasonic atomization in treating chronic sinusitis in 35 cases | not RCT |
| Wang, WP | 2015 | The clinical effect of self xanthium powder combined Sangju decoction in treating 200 cases of nasosinusitis | not RCT |
| Wang, XP | 1996 | The clinical observation of Chinese medicine fumigating nose and internal use on children's chronic maxillary sinusitis | not RCT |
| Wu, LQ | 1996 | The treatment of TCM-WM on Sinusitis for 80 cases | not RCT |
| Josef, H; Fritz, F; Wilhelm, G; et al | 2015 | Traditional Chinese Herbal Medicine in patients with chronic rhinosinusitis - An observational trial comparing the plants' provenance | not RCT |
| Zhu, GP | 1995 | Treating 100 patients with acute suppurative sinusitis by mixture Cang Er Zi | not RCT |
| Ji, HH; Zhou, DW | 1996 | Treating chronic sinusitis with integrated traditional Chinese and western medicine in 62 cases | not RCT |
| Wang, DJ; Ran, Y | 1991 | Treatment of chronic maxillary sinusitis with nasal sinuses perfusate | not RCT |
| Luo, KQ; Yang, JS; Luo, JZ; Wang, GZ | 1994 | Treatment of chronic suppurative maxillary sinusitis with integrated Chinese and western medicine | not RCT |
| Suh, JD; Wu, AW; Taw, MB; Nguyen, C; Wang, MB | 2012 | Treatment of recalcitrant chronic rhinosinusitis with integrative East-West medicine: a pilot study | not RCT |
| Luo, Y; Zhang, C; Zang, C; Gu, S | 2020 | Clinical study of Chinese Medicine fumigation combined with “Zhuyuan Decoction” in the treatment of chronic rhinosinusitis | Outcome NI |
| 施正贤 | 2017 | 中西医结合治疗慢性鼻窦炎及合并眼部疾病临床观察 | Outcome NI |
| 朱耀国; 李日戟; 洪文良; 王练钧 | 1998 | 鼻窦炎汤对鼻窦炎的治疗作用疗效观察 | Outcome NI |
| 王小华 | 2011 | 中西医结合治疗小儿慢性鼻-鼻窦炎49例 | Outcome NI |
| 黄向红 | 2012 | 龙胆泻肝汤加减治疗小儿急性鼻窦炎40例临床观察 | Outcome NI |
| 史凤磊; 周震; 苏秋菊 | 2016 | 中药宣肺通窍颗粒治疗急性鼻窦炎临床观察 | Outcome NI |
| 刘全; 吕传桢 | 2014 | 中西医结合治疗儿童慢性鼻窦炎临床疗效观察 | Outcome NI |
| 雷蕾; 张勉; 陈潇 | 2013 | 中药鼻炎水雾化治疗小儿鼻窦炎临床疗效观察 | Outcome NI |
| 王中霞 | 2009 | 中药治疗儿童慢性鼻窦炎的临床研究 | Outcome NI |
| 杨碧; 赵萍 | 2014 | 玉屏风散加减合阿莫西林治疗鼻窦炎50例临床观察 | Outcome NI |
| 关山越; 李卫红; 罗天飞; 李金奇; 何植洲 | 2009 | 鼻渊汤治疗慢性鼻窦炎临床观察 | Outcome NI |
| 朱优立; 王琳; 马崧 | 2005 | 川芎茶调颗粒冲剂治疗急性鼻窦炎的临床观察 | Outcome NI |
| 丁丽凤 | 2012 | 中西医结合治疗儿童慢性鼻窦炎临床观察 | Outcome NI |
| 宣伟军; 李葵红 | 2000 | 利鼻胶囊治疗儿童慢性上颌窦炎的疗效观察 | Outcome NI |
| 伍梅 | 2006 | 中西医结合治疗慢性鼻窦炎85例临床观察 | Outcome NI |
| 莫凌凌 | 2012 | 香菊胶囊治疗慢性鼻窦炎60例的疗效观察 | Outcome NI |
| 侯自强 | 2009 | 连蒲苍耳子散联合药物冲洗治疗慢性鼻窦炎138例疗效观察 | Outcome NI |
| 可新玲; 马启林 | 2009 | 通窍止痛汤治疗鼻窦炎临床观察90例 | Outcome NI |
| 阙汀贤; 蔡野; 熊琼芳 | 2010 | 自制中药冲洗液治疗慢性鼻窦炎的临床疗效观察 | Outcome NI |
| 田霜 | 2010 | 中西医结合治疗慢性单纯性鼻窦炎疗效观察 | Outcome NI |
| 包秀芝; 罗守信 | 2011 | 鼻渊舒口服液联合布地奈德对慢性鼻窦炎的临床观察 | Outcome NI |
| 陆晓宇 | 2011 | 自拟通鼻汤治疗慢性鼻窦炎临床观察 | Outcome NI |
| 陈扬; 孙海波; 郭少武 | 2012 | 菊花通圣汤治疗急性鼻窦炎(肺经风热型)随机对照临床研究 | Outcome NI |
| 马莉; 刘彬彬; 刘富成 | 2012 | 清鼻丸治疗急性鼻窦炎的疗效观察 | Outcome NI |
| 青淑元, 赵芳芳 | 2012 | 苗药复方鱼鹅滴鼻剂治疗急性鼻窦炎临床研究 | Outcome NI |
| 余亚明 | 2012 | 藿胆丸配合鼻腔冲洗治疗慢性鼻窦炎68例 | Outcome NI |
| 周晖; 王小晖; 郭鸿; 李红艳; 李成君; 倪富强 | 2012 | 鼻渊舒口服液治疗慢性鼻窦炎150例临床观察 | Outcome NI |
| 邵瑜 | 2013 | 鼻渊舒口服液治疗鼻窦炎的临床效果 | Outcome NI |
| 黄瑛 | 2014 | 鼻渊通窍颗粒联合阿莫西林治疗急性鼻窦炎随机平行对照研究 | Outcome NI |
| 房耿浩 | 2015 | 解郁祛湿建中汤治疗湿热型慢性鼻窦炎临床观察 | Outcome NI |
| 刘宪宾 | 2015 | 鼻渊舒口服液对慢性鼻窦炎的辅助治疗效果分析 | Outcome NI |
| 庞敏飞 | 2015 | 苍耳鼻窦炎方联合头孢米诺钠治疗化脓性鼻窦炎临床观察 | Outcome NI |
| 李头清 | 2016 | 中西医结合治疗慢性鼻窦炎30例 | Outcome NI |
| 佘文胜; 皮本元 | 2016 | 鱼腥草鼻用喷雾剂联合羟甲唑啉鼻喷雾剂、头孢丙烯治疗急性鼻窦炎随机对照研究 | Outcome NI |
| 高东延 | 2017 | 注射用双黄连鼻腔冲洗治疗慢性鼻-鼻窦炎的疗效分析 | Outcome NI |
| 唐爱华 | 2017 | 鼻渊汤随证加减联合罗红霉素治疗急性鼻窦炎的疗效和安全性分析 | Outcome NI |
| 吴小海; 赵丽华; 张奕; 程靖; 艾力 | 2017 | 裸花紫珠片治疗儿童急性鼻窦炎的疗效观察 | Outcome NI |
| 马成; 刘炜明; 杨光远; 王永国 | 2018 | 验方鼻窦炎丸治疗慢性鼻窦炎的临床研究 | Outcome NI |
| 张杨; 郭洁 | 2018 | 中西药合用治疗慢性鼻窦炎胆腑郁热证疗效观察 | Outcome NI |
| 王宗杰 | 2010 | 中药鼻渊冲剂治疗急性鼻窦炎的临床疗效观察 | Outcome NI |
| 仲崇玉 | 2012 | 辛银鼻窦汤结合两药治疗慢性鼻窦炎疗效观察 | Outcome NI |
| 杨正佳 | 2013 | 加减龙胆泻肝冲剂治疗胆腑郁热型鼻渊的临床观察 | Outcome NI |
| 杨宁; 林清霞; 何瑞华; 吕八铎 | 2014 | 鼻渊舒口服液联合罗红霉素治疗慢性鼻窦炎的疗效观察 | Outcome NI |
| 董艳玲 | 2016 | 鼻渊软胶囊联合罗红霉素治疗慢性鼻窦炎的临床效果 | Outcome NI |
| 张书龙; 苟再菊 | 2016 | 慢性鼻-鼻窦炎中西医结合治疗的效果观察 | Outcome NI |
| 艾合买提, 木合塔尔 | 2020 | 温肺止流丹加减治疗肺气虚寒型慢性鼻-鼻窦炎（Ⅰ型）的临床疗效观察 | Outcome NI |
| 汤楠 | 2020 | 中西药合用治疗慢性鼻窦炎肝胆湿热郁结型临床观察 | Different endpoint for treatment group and control group |
| 刘国新 | 2018 | 千金苇茎汤联合西药治疗鼻窦炎(痰瘀阻滞)随机平行对照研究 | Outcome NI |
| 刘增 | 2019 | 鼻渊软胶囊联合头孢呋辛治疗急性鼻窦炎的临床疗效观察 | Outcome NI |
| 邱红艳 | 2019 | 银黄清肺胶囊联合阿奇霉素治疗肺经风热型急性鼻窦炎临床研究 | Outcome NI |
| 汪娟 | 2019 | 中西药合用治疗急性鼻窦炎临床观察 | Outcome NI |
| 张忆辰 | 2019 | 清化饮治疗中焦湿热型鼻窦炎的临床疗效观察 | CHM 1 vs CHM 2 |
| 朱慧贤 | 2019 | 鼻渊丸治疗急性鼻窦炎风邪犯肺证的多中心临床研究 | CHM 1 vs CHM 2 |
| 庚新 | 2019 | 温肺通窍汤治疗肺气虚寒型儿童慢性鼻—鼻窦炎的临床观察 | Outcome NI |
| 何春玲 | 2019 | 中西医结合治疗儿童慢性鼻窦炎疗效观察 | Outcome NI |
| 钱丹 | 2019 | 龙胆泻肝汤加减治疗儿童慢性鼻窦炎肝胆湿热证临床研究 | Outcome NI |
| 宋晓 | 2020 | 益气通窍汤鼻窦冲洗联合雾化治疗慢性鼻窦炎患儿的效果 | Outcome NI |
| 孙嘉蔚 | 2019 | 中西医结合治疗儿童慢性鼻-鼻窦炎临床观察 | Outcome NI |
| 王梦奇 | 2019 | 运脾祛湿清热方治疗小儿急性鼻窦炎湿热证的临床观察 | Outcome NI |
| 姚期 | 2019 | 用参苓白术散合苍耳子散对慢性化脓性鼻窦炎患儿进行治疗的效果分析 | Outcome NI |
| 于凤英 | 2020 | 解表清里法联合西药治疗儿童急性鼻窦炎的疗效观察 | Outcome NI |
| 张勉 | 2019 | 参苓白术散加减治疗小儿鼻窦炎脾虚湿盛证临床观察 | Outcome NI |
| 陈波 | 2019 | 自拟清宣鼻窍饮经验方治疗风热壅肺型慢性鼻窦炎急性发作期患儿38例 | Unclear Diagnosis |
| 张玉丽;蔚金建;康犁阳 | 2022 | 中西医结合阶段性治疗儿童慢性鼻-鼻窦炎的临床研究 | Outcome NI |
| 张伟;李志钢 | 2021 | 中药高容量冲洗治疗对FESS术后慢性鼻—鼻窦炎患者鼻黏膜炎症干预的临床观察 | Outcome NI |
| 艾斌;齐建强 | 2021 | 中药鼻腔雾化治疗慢性鼻窦炎的相关研究 | Outcome NI |
| 彭波 | 2021 | 鼻内镜手术后中药鼻腔冲洗治疗慢性鼻窦炎患者的效果 | Retrospective study, not RCT |
| 谭畅;张利丹;支英杰;谢雁鸣 | 2022 | 鼻渊通窍颗粒治疗慢性鼻窦炎有效性和安全性的系统评价与Meta分析 | Systematic review |
| 林曼青;周敏;陈腾宇;李丹;方彩珊;王睿智;朱锦祥;阮岩;徐慧贤;王培源 | 2022 | 中药鼻腔冲洗治疗慢性鼻窦炎术后有效性和安全性的Meta分析 | Systematic review |
| 刘峘;崔鑫;谢雁鸣;黎元元 | 2022 | 香菊胶囊治疗慢性鼻-鼻窦炎的药物经济学评价 | Systematic review |
| 张耀军;江燕;胡慧娟;齐银辉;王中霞;于洁 | 2022 | 慢性鼻-鼻窦炎中西医治疗研究进展 | Review article |

Abbreviations: CHM: Chinese herbal medicine; Outcome NI: outcome measures were not included in this review; RCT: Randomized controlled trial; vs: versus.

**S3 Table. Ingredients of the CHM interventions, manufacture and dosage used in the included studies and funding**

| **Study name (funding)** | **CHM intervention; Manufacturer; Dosage** | **Control intervention; manufacturer; Dosage** | **Ingredients of CHMs: Latin binomial (Chinese name in pinyin)^1^** |
| --- | --- | --- | --- |
| Cai CJ 2019  (NA) | *Gan lu xiao du dan* (甘露消毒丹) GLXDD; decoction, prepared on site at Fourth Affiliated Hospital of Traditional Chinese Medicine of Xinjiang Medical University, Xinjiang, China (中药汤剂由新疆医科大学第四附属中医医院药房煎制及分装、发放); 200ml per dose, twice daily | mometasone furoate nasal spray; Merck & Co., Inc.(默沙东药公司); 2 sprays each side, once daily | *Pogostemon cablin* (Blanco) Benth. (huo xiang), Artemisia *scoparia* Waldst. et Kit. (yin chen), *Forsythia suspensa* (Thunb.) Vahl (lian qiao), *Hydrated magnesium silicate (hua shi), Amomum krarvanh* Pierre ex Gagnep (bai kou ren), *Acorus tatarinowii* Schott (shi chang pu), *Acorus tatarinowii* Schott (bei mu), *Mentha haplocalyx* Briq. (bo he), *Iris tectorum* Maxim. (she gan), *Clematis armandii* Franch. (chuan mu tong), *Scutellaria baicalensis* Georgi (huang qin) |
| Chen TT 2017  (NA) | *Long dan xie gan tang* (龙胆泻肝汤) LDXGT; decoction, prepared on site at Weifang people’s hospital, Shandong, China (潍坊市人民医院, 受试者服用的汤剂由专业人员统一煎煮); 1 sachet 3 times daily | oral clarithromycin; NS; 250mg daily taken with food plus mometasone furoate nasal spray 1 spray each side, once daily | *Gentiana manshurica* Kitage (long dan cao), *Scutellaria baicalensis* Georgi (huang qin), *Gardenia jasminoides* Elli (zhi zi), *Alisma orientalis* (Sam.) Juzep (ze xie), *Clematis armandii* Franch (chuan mu tong), *Plantago asiatica* L (che qian zi), *Angelica sinensis* (Oliv.) Diel (dang gui), *Rehmannia glutinosa* Libosch (di huang), *Bupleurum chinense* DC (chai hu), *Glycyrrhiza uralensis* Fisch (gan cao) |
| Chu XY 2017 (NA) | *Bi yuan tong qiao ke li* (鼻渊通窍颗粒) BYTQKL, granules; Lunan Pharmaceutical Group Corporation, China. State medical permit no: Z20030071 (山东新时代药业有限公司, 国药准字Z20030071); 1 sachet per dose, 3 times daily plus the same pharmacotherapy | triamcinolone acetonide nasal spray; Kunming Yuanrui Pharmacy Limited Company, China. State medical permit no: H20051175 (昆明源瑞制药有限公司, 国药准字H20051175); 220μg daily | *Ephedra sinica* Stapf (ma huang), *Xanthium sibiricum* Patr (cang er zi), *Magnolia biondii* Pamp (xin yi), *Angelica dahurica* (Fisch. ex Hoffm.) Benth. et Hook. f (bai zhi), *Scutellaria baicalensis* Georgi (huang qin), *Poria cocos* (Schw.) Wolf (fu ling), *Mentha haplocalyx* Briq (bo he), *Ligusticum sinense* Oliv (gao ben), *Chrysanthemum indicum* L (ye ju hua), *Rehmannia glutinosa* Libosch (di huang) |
| Dai RZ 2015 (1) | *Bi yuan shu kou fu ye* (鼻渊舒口服液) BYSKFY, vial; Chengdu Huasun Pharmaceutical Co. Ltd., China. (成都华神集团股份有限公司制药厂生产); one 10 ml vial per dose, 3 times daily | oral clarithromycin capsules; Lizhu Group Lizhu Pharmaceutical Factory, China (丽珠集团丽珠制药厂); 0.25g twice daily | *Scutellaria baicalensis* Georgi (huang qin), *Gentiana manshurica* Kitage (long dan cao), *Angelica dahurica* (Fisch. ex Hoffm.) Benth. et Hook. f (bai zhi), *Xanthium sibiricum* Patr (cang er zi), *Magnolia biondii* Pamp (xin yi), *Schizonepeta tenuifolia* Briq (jing jie), *Mentha haplocalyx* Briq (bo he), *Bupleurum chinense* DC (chai hu), *Astragalus membranaceus* (Fisch.) Bge. *var. mongholicus* (Bge.) Hsiao (huang qi), *Ligusticum chuanxiong* Hort (chuang xiong), *Clematis armandii* Franch (chuan mu tong) |
| Deng QH 2016 (NA) | *Bi yuan tong qiao ke li* (鼻渊通窍颗粒) BYTQKL, granules; NS; 15g per dose, 3 times daily | triamcinolone acetonide nasal spray; NS; 220μg twice daily | *Magnolia biondii* Pamp (xin yi), *Xanthium sibiricum* Patr (cang er zi), *Ephedra sinica* Stapf (ma huang), *Angelica dahurica* (Fisch. ex Hoffm.) Benth. et Hook. f (bai zhi), *Mentha haplocalyx* Briq (bo he), *Ligusticum sinense* Oliv (gao ben), *Scutellaria baicalensis* Georgi (huang qin), *Poria cocos* (Schw.) Wolf (fu ling*), Rehmannia glutinosa* Libosch (di huang), *Chrysanthemum indicum* L (ye ju hua), *Forsythia suspensa* (Thunb.) Vahl (lian qiao) |
| Du JW 2016 (NA) | *Bi dou yan kou fu ye* (鼻窦炎口服液) BDYKFY, vial; Chongqing Taiji Industry (Group) Co., Ltd., China. State medical permit no: Z50020160; one 10 ml vial per dose, 3 times daily | oral clarithromycin capsules; State medical permit no: H20067196; (国药准字号 H20067196; 0.25g twice daily | *Schizonepeta tenuifolia* Briq (jing jie), *Magnolia biondii* Pamp (xin yi), *Xanthium sibiricum* Patr (cang er zi), *Angelica dahurica* (Fisch. ex Hoffm.) Benth. et Hook. f (bai zhi), *Bupleurum chinense* DC (chai hu), *Scutellaria baicalensis* Georgi (huang qin), *Ligusticum chuanxiong* Hort (chuang xiong), *Platycodon grandiflorum* (Jacq.) A. DC (jie geng), *Gardenia jasminoides* Elli (zhi zi), *Gentiana manshurica* Kitage (long dan cao), *Astragalus membranaceus* (Fisch.) Bge. var. mongholicus (Bge.) Hsiao (huang qi) |
| Guo L 2015 (NA) | *Bi yan kang tang* (鼻炎康汤) BYKT, decoction; patients cook by themselves; one packet per day, taken as two doses; plus another packet of the same CHM decocted and used as a nasal wash, 100 ml per side, twice daily for 4wks, then, both groups received half the dose of clarithromycin for a further 8 weeks as a maintenance treatment | oral clarithromycin; Shandong Xinhua pharmaceutical Co. Ltd, China. State medical permit no: H20000354 (山东新华制药股份有限公司, 国药准字H20000354); 250 mg, two times daily, plus a nasal wash 100 ml per side, twice daily composed of 0.9% saline plus plus dexamethasone for 4wks, then, both groups received half the dose of clarithromycin for a further 8 weeks as a maintenance treatment | *Angelica sinensis* (Oliv.) Diel (dang gui), *Ligusticum chuanxiong* Hort (chuang xiong*), Rehmannia glutinosa* Libosch (di huang), *Acorus tatarinowii* Schott (shi chang pu), *Angelica dahurica* (Fisch. ex Hoffm.) Benth. et Hook. f (bai zhi), *Lonicera japonica* Thunb (jin yin hua), *Chrysanthemum morifolium* Ramat (ju hua), *Taraxacum mongolicum* Hand. Mazz (pu gong ying), *Gardenia jasminoides* Elli (zhi zi), *Glycyrrhiza uralensis* Fisch (gan cao), *Schizonepeta tenuifolia* Briq (jing jie), *Saposhnikovia divaricata* (Turcz.) Schischk (fang feng), *Asarum heterotropoides* Fr. Schmidt *var. mandshuricum* (Maxim) Kitag (xi xin), *Magnolia biondii* Pamp (xin yi), *Xanthium sibiricum* Patr (cang er zi) |
| Hong HY 2015 (2) | *Bi yuan tong qiao ke li* (鼻渊通窍颗粒) BYTQKL, granules; Lunan Pharmaceutical Group Corporation, China. State medical permit no: Z20030071 (山东鲁南制药股份有限公司, Z20030071); 15g per dose, 3 times daily | oral clarithromycin; Abbott Laboratories. State medical permit no: H20033044 (雅培制药有限公司 H20033044); 0.25g twice daily | *Magnolia biondii* Pamp (xin yi), *Angelica dahurica* (Fisch. ex Hoffm.) Benth. et Hook. f (bai zhi), *Xanthium sibiricum* Patr (cang er zi), *Ephedra sinica* Stapf (ma huang), *Ligusticum sinense* Oliv (gao ben), *Forsythia suspensa* (Thunb.) Vahl (lian qiao), *Scutellaria baicalensis* Georgi (huang qin), *Trichosanthes kirilowii* Maxim (tian hua fen), *Mentha haplocalyx* Briq (bo he), *Salvia miltiorrhiza* Bge (dan shen), *Rehmannia glutinosa* Libosch (di huang), *Chrysanthemum indicum* L (ye ju hua), *Poria cocos* (Schw.) Wolf (fu ling), *Glycyrrhiza uralensis* Fisch (gan cao) |
| Hu FL 2015 (NA) | *Bi yuan shu kou fu ye* (鼻渊舒口服液) BYSKFY, vial; Chengdu Huasun Pharmaceutical Co. Ltd., China. State medical permit no: Z51020208 (成都华神集团股份有限公司制药厂, 国药准字Z51020208, 规格为每支10 mL); one 10 ml vial per dose, 3 times daily | oral clarithromycin; Yatai Pharma, China. State permit medical no: H20058223 (浙江亚太药业股份有限公司, 国药准字H20058223, 规格为每粒0.125 g); one 0.125 g capsule twice daily | *Magnolia biondii* Pamp (xin yi), *Xanthium sibiricum* Patr (cang er zi), *Scutellaria baicalensis* Georgi (huang qin), *Bupleurum chinense* DC (chai hu), *Angelica dahurica* (Fisch. ex Hoffm.) Benth. et Hook. f (bai zhi), *Astragalus membranaceus* (Fisch.) Bge. *var. mongholicus* (Bge.) Hsiao (huang qi) |
| Huang JY 2017 (NA) | *Bi dou yan kou fu ye* (鼻窦炎口服液) BDYKFY, vial; Chongqing Taiji Industry (Group) Co., Ltd., China (太极集团重庆桐君阁药厂有限公司, 10 mL/支); one 10 ml vial, 3 times daily | oral cefuroxime tablets; Shenzhen Zhijun Pharmaceutical Co., Ltd., China. (深圳致君制药有限公司, 0.25 g/片); 0.25g twice daily | *Magnolia biondii* Pamp (xin yi)*, Schizonepeta tenuifolia* Briq (jing jie), *Mentha haplocalyx* Briq (bo he), *Platycodon grandiflorum* (Jacq.) A. DC (jie geng), *Bupleurum chinense* DC (chai hu), *Xanthium sibiricum* Patr (cang er zi), *Angelica dahurica* (Fisch. ex Hoffm.) Benth. et Hook. f (bai zhi), *Ligusticum chuanxiong* Hort (chuang xiong), *Scutellaria baicalensis* Georgi (huang qin), *Gardenia jasminoides* Elli (zhi zi), *Poria cocos* (Schw.) Wolf (fu ling), *Clematis armandii* Franch (chuan mu tong), *Astragalus* membranaceus (Fisch.) Bge. *var. mongholicus* (Bge.) Hsiao (huang qi), *Gentiana manshurica* Kitage (long dan cao) |
| Jiang RS 2012 (NA) | *Cang er zi san* (苍耳子散) CEZS modified, capsules; NS; 2 capsules (1 for placebo of erythromycin, 1 for CHM) every 12 hours | erythromycin plus a placebo for the CHM; NS; 2 capsules (1 placebo for CHM, 1 for erythromycin) every 12 hours | *Xanthium sibiricum* Patr (cang er zi), *Magnolia biondii* Pamp (xin yi), *Angelica dahurica* (Fisch. ex Hoffm.) Benth. et Hook. f (bai zhi), *Mentha haplocalyx* Briq (bo he), *Houttuynia cordata* Thunb (yu xing cao) |
| Liao WT 2020 (NA) | *Bi yan kang tang* (鼻炎康汤) BYKT, decoction, cooked by patients themselves; NS; one bag per day, 400ml per dose, twice daily | oral clarithromycin; Jiangsu Hengrui Medicine Company Ltd., China. Lot no: 1902204 (江苏恒瑞医药股份有限公司, 批号1902204, 0.5g/片); 0.5g once daily; plus, gentamicin sulfate injection 80mg (Guangdong Sancai Shiqi Pharmaceutical Co., Ltd., China. Lot no: 190330 广东三才石岐制药股份有限公司, 批号190330) + dexamethasone sodium phosphate injection 20mg (Tianjin KingYork Group Hubei Tianyao Pharmaceutical Co., Ltd, China. Lot no: 1904209 天津金耀集团湖北天药药业股份有限公司, 批号1904209) + normal saline; nasal wash, 100ml each side, twice daily | *Angelica sinensis* (Oliv.) Diel (dang gui), *Ligusticum chuanxiong* Hort (chuang xiong*), Rehmannia glutinosa* Libosch (di huang), *Acorus tatarinowii* Schott (shi chang pu), *Angelica dahurica* (Fisch. ex Hoffm.) Benth. et Hook. f (bai zhi), *Lonicera japonica* Thunb (jin yin hua), *Chrysanthemum morifolium* Ramat (ju hua), *Taraxacum mongolicum* Hand. Mazz (pu gong ying), *Gardenia jasminoides* Elli (zhi zi), *Glycyrrhiza uralensis* Fisch (gan cao), *Schizonepeta tenuifolia* Briq (jing jie), *Saposhnikovia divaricata* (Turcz.) Schischk (fang feng), *Asarum heterotropoides* Fr. Schmidt *var. mandshuricum* (Maxim) Kitag (xi xin), *Magnolia biondii* Pamp (xin yi), *Xanthium sibiricum* Patr (cang er zi) |
| Li MJ 2014a, b (NA) | *Cang er zi san* (苍耳子散) CEZS modified;  a. decoction; cooked by patients themselves; NS.  b. oral + nasal *Cang er zi san* 苍耳子散 decoction was used as an inhalation and then consumed orally for one week and in the following week the same decoction was used as an inhalation only; NS | c. oral amoxicillin; NS; 2 tablets twice daily | *Xanthium sibiricum* Patr (cang er zi), *Magnolia biondii* Pamp (xin yi), *Angelica dahurica* (Fisch. ex Hoffm.) Benth. et Hook. f (bai zhi), *Mentha haplocalyx* Briq (bo he), *Scutellaria baicalensis* Georgi (huang qin), *Ligusticum chuanxiong* Hort (chuang xiong), *Platycodon grandiflorum* (Jacq.) A. DC (jie geng), *Thlaspi arvense* Linn (bai jiang cao) |
| Lin L 2015 (NA) | *Lian hua qing wen ke li* (连花清瘟颗粒) LHQWKL, granules; Shijiazhuang Yiling Pharmaceutical Co. Ltd., China (石家庄以岭药业股份有限公司); 1 sachet 3 times daily | a. no treatment;  b. oral amoxicillin capsules; United Laboratories, Zhuhai, China. (珠海联邦制药股份有限公司); 1000mg 3 times daily;  c. rhinocort nasal spray; AstraZeneca; 128 microg daily | *Forsythia suspensa* (Thunb.) Vahl (lian qiao), *Lonicera japonica* Thunb (jin yin hua), *Ephedra sinica* Stapf (ma huang), *Hydrated calcium* sulphate (shi gao*), Rheum palmatum* L (da huang), *Rhodiola crenulata* (Hook. f. et Thoms.) H. Ohba *Glycyrrhiza uralensis* Fisch (gan cao), etc |
| Lin L 2020  (NA) | *Lian hua qing wen ke li* (连花清瘟颗粒) LHQWKL, granules; 13 herbs  Beijing Yiling Pharmaceutical Co., Ltd, Chinese traditional medicine Quasiword, Z20100040; production batch, 1710002; 6 g/packet, 1 packet 3 times daily | Placebo; Beijing Yiling Pharmaceutical Co., Ltd, Chinese traditional medicine Quasiword, Z20100040; production batch, 1712033; 6 g/packet, 1 packet 3 times daily. The raw material of placebo only comprises 1 ingredient (*Lonicera confusa* DC.) according to Pharmacopoeia of People's Republic of China (Version 2010). | *Dryopteris crassirhizoma* Nakai (guan zhong*), Houttuynia cordata* Thunb. (yu xing cao)*, Lonicera japonica* Thunb. (jin yin hua), *Forsythia suspensa* (Thunb.) Vahl (lian qiao), *Gypsum Fibrosum* (shi gao), *Isatis indigotica* Fortune (ban lan gen)*, Ephedra sinica* Stapf (ma huang), *Rhodiola rosea* L. (hong jing tian), *Armeniaca sibirica* (L.) Lam. (xing ren), *Glycyrrhiza uralensis* Fisch. (gan cao), *Pogostemon cablin* (Blanco) Benth. (huo xiang)*, Rheum palmatum* L. (da huang)*, Mentha haplocalyx* Briq. (bo he) |
| Liu HL 2017 CHMa (3) | *Bi yuan shu wan* (鼻渊舒丸) BYSW, pill; prepared on site at First Affiliated Hospital of Hunan University of Chinese Medicine, Hunan, China (由湖南中医药大学第一附属医院药剂科制成丸剂, 每瓶100 g); 8g per dose, 3 times daily | oral eucalyptol, limonene and pinene enteric soft capsules; Beijing Jiuhe Pharmaceutical Co., Ltd. China. (北京九和药业有限公司); one 0.3g capsule twice daily | *Astragalus membranaceus* (Fisch.) Bge. *var. mongholicus* (Bge.) Hsiao (huang qi), *Codonopsis pilosula* (Franch.) Nannf (dang shen), *Dioscorea opposita* Thunb (shan yao), *Poria cocos* (Schw.) Wolf (fu ling), *Platycodon grandiflorum* (Jacq.) A. DC (jie geng), *Angelica dahurica* (Fisch. ex Hoffm.) Benth. et Hook. f (bai zhi), *Magnolia biondii* Pamp (xin yi), *Scutellaria baicalensis* Georgi (huang qin), *Gleditsia sinensis* Lam (zao jiao ci), *Acorus tatarinowii* Schott (shi chang pu), *Glycyrrhiza uralensis* Fisch (gan cao) |
| Liu HL 2017 CHMb (3) | *Tong qiao bi yan ke li* (通窍鼻炎颗粒) TQBYKL, granules; Sichuan Chuanda Huaxi pharmaceutical Co., Ltd., Sichuan, China (四川川大华西药业股份有限公司); one 2g sachet per dose, 3 times daily | oral eucalyptol limonene and pinene enteric soft capsules; Beijing Jiuhe Pharmaceutical Co., Ltd. China (北京九和药业有限公司); one 0.3g capsule twice daily | *Xanthium sibiricum* Patr (cang er zi), *Saposhnikovia divaricata* (Turcz.) Schischk (fang feng), *Astragalus membranaceus* (Fisch.) Bge. *var. mongholicus* (Bge.) Hsiao (huang qi), *Angelica dahurica* (Fisch. ex Hoffm.) Benth. et Hook. f (bai zhi), *Magnolia biondii* Pamp (xin yi), *Atractylodes macrocephala* Koidz (bai zhu) |
| Liu JB 2011 (4) | *Bi yuan shu jiao nang* (鼻渊舒胶囊) BYSJN, capsules; Chengdu Huashen Group Co., Ltd., Sichuan, China (成都华神集团股份有限公司制药厂, 0.3g /粒); 3 capsules 3 times daily for the first two weeks, then twice daily for the next two weeks, and once daily for the final 8 weeks of the 12-week study | oral clarithromycin; Guangdong Huideqin Pharmaceutical Co., Ltd., China (广东惠德勤药业有限公司); 0.25g tablet, twice daily for the first two weeks, then once daily for the next 10 weeks | *Xanthium sibiricum* Patr (cang er zi), *Magnolia biondii* Pamp (xin yi), *Mentha haplocalyx* Briq (bo he), *Angelica dahurica* (Fisch. ex Hoffm.) Benth. et Hook. f (bai zhi), *Scutellaria baicalensis* Georgi (huang qin), *Gardenia jasminoides* Elli (zhi zi), *Bupleurum chinense* DC (chai hu), *Asarum heterotropoides* Fr. Schmidt *var. mandshuricum* (Maxim) Kitag (xi xin), *Ligusticum chuanxiong* Hort (chuang xiong), *Astragalus membranaceus* (Fisch.) Bge. *var. mongholicus* (Bge.) Hsiao (huang qi*), Clematis armandii* Franch (chuan mu tong), *Platycodon grandiflorum* (Jacq.) A. DC (jie geng) |
| Liu Q 2015 (NA) | *Bi dou yan kou fu ye* (鼻窦炎口服液) BDYKFY; vial; NS; one 10 ml vial per dose, twice daily | oral clarithromycin; NS; 250 mg once a day | *Magnolia biondii* Pamp (xin yi), *Astragalus membranaceus* (Fisch.) Bge. *var. mongholicus* (Bge.) Hsiao (huang qi), *Platycodon grandiflorum* (Jacq.) A. DC (jie geng), *Clematis armandii* Franch (chuan mu tong), *Bupleurum chinense* DC (chai hu), *Angelica dahurica* (Fisch. ex Hoffm.) Benth. et Hook. f (bai zhi), *Xanthium sibiricum* Patr (cang er zi), *Ligusticum chuanxiong* Hort (chuan xiong), *Poria cocos* (Schw.) Wolf (fu ling), *Schizonepeta tenuifolia* Briq (jing jie), *Scutellaria baicalensis* Georgi (huang qin), *Gardenia jasminoides* Elli (zhi zi), *Mentha haplocalyx* Briq (bo he), *Gentiana manshurica* Kitage (long dan cao) |
| Qiang JH 2011a (NA) | *Bi yuan he ji* (鼻渊合剂) BYHJ, decoction, prepared on site at Jiangsu Hospital of Traditional Chinese Medicine, Jiangsu, China; (药物由我院精制加工制成1000 mL药汁); 20 ml per dose, twice daily | erythromycin enteric-coated capsules; Zhejiang Lishui Zhongyi Pharmaceutical Industry Limited Company, China. State medical permit no: H20065180 (浙江丽水众益药业有限公司, 国药准字H20065180); 250mg, twice daily | *Cynanchum paniculatum* (Bge.) Kitag. (xu chang qing), *Taraxacum mongolicum* Hand. -Mazz. (pu gong ying), *Benincasa hispida* (Thunb.) Cogn. (dong gua zi), Morus alba L. (sang ye), *Angelica dahurica* (Fisch. ex Hoffm.) Benth. et Hook. f. (bai zhi), *Acorus tatarinowii* Schott (shi chang pu), *Magnolia biondii* Pamp (xin yi), *Glycyrrhiza uralensis* Fisch (gan cao) |
| Qiang JH 2011b (NA) | *Bi yan pian* (鼻炎片) BYP, pills; Wuhan Zhonglian Pharmaceutical Group Co., Ltd. China. State medical permit no: Z42021537 (武汉中联药业集团股份有限公司, 国药准字Z42021537); 2 pills per dose, 3 times daily | As above | *Xanthium sibiricum* Patr (cang er zi), *Magnolia biondii* Pamp (xin yi), *Saposhnikovia divaricata* (Turcz.) Schischk (fang feng), *Forsythia suspensa* (Thunb.) Vahl (lian qiao), *Chrysanthemum indicum* L (ye ju hua), *Schisandra chinensis* Baill (wu wei zi), *Platycodon grandiflorum* (Jacq.) A. DC. (jie geng), *Angelica dahurica* (Fisch. ex Hoffm.) Benth. et Hook. f. (bai zhi), *Anemarrhena asphodeloides* Bunge (zhi mu), *Schizonepeta tenuifolia* Briq (jing jie), *Glycyrrhiza uralensis* Fisch (gan cao) |
| Wang C 2014 (NA) | *Bi yuan shu kou fu ye* (鼻渊舒口服液) BYSKFY, vial; Chengdu Huasun Pharmaceutical Co. Ltd., China. State medical permit no: Z51020208 (成都华神集团股份有限公司制药厂, 国药准字Z51020208, 10 mL/支); one 10 ml vial per dose, twice daily | oral clarithromycin; Jiangsu Hengrui Medicine Pharmaceutical company, China. State medical permit no: H20031041 (江苏恒瑞医药股份有限公司, 国药准字H20031041, 规格: 0.5 克/片, 批号 20130403); 500 mg once a day (taken with food) plus oral eucalyptol, limonene and pinene enteric soft capsules (Myrtol®); Germany Porsche Grand Pharmaceutical Factory. State permit medical no: Z20030020 (德国保时佳大药厂, 国药准字Z20030020, 规格mg /粒, 批号20130221); 0.3g, 3 times daily, plus a nasal wash of 500 ml saline once a day | *Magnolia biondii* Pamp (xin yi), *Xanthium sibiricum* Patr (cang er zi), *Scutellaria baicalensis* Georgi (huang qin), *Bupleurum chinense* DC (chai hu), *Astragalus membranaceus* (Fisch.) Bge*. var. mongholicus* (Bge.) Hsiao (huang qi) |
| Wang G 2013 (NA) | *Ma yi bi yan pen wu ji* (麻夷鼻炎喷雾剂) MYBYPWJ, nasal spray; State medical permit no: H20104201 (国药准字: H20104201); 2 sprays each side of nose, 3 times daily | oral cefixime or roxithromycin; NS; at routine doses | *Ephedra sinica* Stapf (ma huang), *Magnolia biondii* Pamp (xin yi), *Asarum heterotropoides* Fr. Schmidt *var. mandshuricum* (Maxim) Kitag (xi xin*), Phellodendron chinense* Schneid (huang bai), Borneol (bing pian), Camphor (zhang nao) |
| Wang H 2009 | *Bi yuan gu ben fang* (鼻渊固本方) BYGBF, decotion; NS; 50ml, 3 times daily | oral cefadroxil; CSPC Ouyi Pharmaceutical Co., Ltd. China. State medical permit no: H10960160 (石药集团欧意药业有限公司生产, 国药准字H10960160); 30 mg/kg per day, twice daily.  nasal spray of chloramphenicol + dexamethasone; CSPC Zhongnuo Pharmaceutical Co., Ltd. Shijiazhuang, China. State medical permit no: H13021662 (石药集团中诺药业(石家庄)有限公司, 国药准字H13021662); 1 spray each side, twice daily | *Astragalus membranaceus* (Fisch.) Bge. *var. mongholicus* (Bge.) Hsiao (huang qi), *Codonopsis pilosula* (Franch.) Nannf (dang shen), *Poria cocos* (Schw.) Wolf (fu ling), *Magnolia biondii* Pamp (xin yi), *Angelica dahurica* (Fisch. ex Hoffm.) Benth. et Hook. f. (bai zhi), *Platycodon grandiflorum* (Jacq.) A. DC. (jie geng), *Xanthium sibiricum* Patr (cang er zi), *Cynanchum paniculatum* (Bge.) Kitag (xu chang qing), *Scutellaria baicalensis* Georgi (huang qin), *Glycyrrhiza uralensis* Fisch (gan cao) |
| Wang J 2020 (NA) | *Yu jiang pai du he ji* (鱼酱排毒合剂) YJPDHJ, nasal wash; Yongchuan Hospital of Traditional Chinese Medicine Affiliated to Chongqing Medical University, China (重庆医科大学附属永川中医院院内制剂) 20ml/time, twice daily | 0.9% Saline; NS; 20ml/time, twice daily | *Houttuynia cordata* Thunb (yu xing cao), *Patrinia villosa* (Thunb.) Juss. (bai jiang cao), *Xanthium sibiricum* Patr (cang er zi), *Magnolia biondii* Pamp (xin yi), *Ligusticum chuanxiong* Hort. (chuan xiong), *Angelica dahurica* (Fisch. ex Hoffm.) Benth. et Hook. f (bai zhi), Gypsum fibrosum (sheng shi gao), *Scutellaria baicalensis* Georgi (huang qin), *Gleditsia sinensis* Lam. (zao jiao ci), *Glycyrrhiza uralensis* Fisch (gan cao) etc |
| Wang KQ 2016 (NA) | *Bi yuan tang* (鼻渊汤) BYT1, nasal wash; prepared on site at Tibet Military General Hospital, Tibet, China (冲洗液均是由我院制剂室进行统一煎制-西藏军区总医院耳鼻喉科, 西藏拉萨); 100ml each side of nose, twice daily | oral cefaclor tablets; Lilly Suzhou Pharmaceutical Co., Ltd., China. Lot no: 20140204 (礼来苏州制药有限公司, 规格: 0. 125g /袋, 生产批号: 20140204); 0.25g, 3 times daily plus budesonide nasal spray; AstraZeneca Pharmaceuticals. Lot no: 20140202 (阿斯利康制药有限公司; 规格: 64μg 120 喷; 生产批号: 20140202); twice daily | *Magnolia biondii* Pamp (xin yi), *Xanthium sibiricum* Patr (cang er zi), *Acorus tatarinowii* Schott (shi chang pu), *Houttuynia cordata* Thunb (yu xing cao*), Centipeda minima* (L.) A. Br. et Aschers (e bu shi cao), *Angelica dahurica* (Fisch. ex Hoffm.) Benth. et Hook. f (bai zhi), *Poria cocos* (Schw.) Wolf (fu ling), *Astragalus membranaceus* (Fisch.) Bge. *var. mongholicus* (Bge.) Hsiao (huang qi), *Alisma orientalis* (Sam.) Juzep (ze xie) |
| Wang P 2015 (NA) | *Xin zhi di bi ye* (辛芷滴鼻液) XZDBY, nasal drop; prepared on site at Jiugang Hospital, Gansu, China (酒钢医院耳鼻咽喉科); 3 drops each side of nose, 3 times daily | oral roxithromycin; NS; 0.15g once a day plus fluticasone nasal spray; NS; 1 spray each side once a day | *Magnolia biondii* Pamp (xin yi), *Xanthium sibiricum* Patr (cang er zi), *Saposhnikovia divaricata* (Turcz.) Schischk (fang feng), *Houttuynia cordata* Thunb (yu xing cao), *Scutellaria baicalensis* Georgi (huang qin), *Prunella vulgaris* L (xia ku cao), *Angelica dahurica* (Fisch. ex Hoffm.) Benth. et Hook. f (bai zhi), etc |
| Wu MM 2022 (5) | *Tong bi xiao ti ke li* (通鼻消涕颗粒) TBXTKL, granules; prepared on site at Yunnan Traditional Chinese Medicine Hospital, China (云南省中医医院院内制剂); 15g per dose, twice daily | oral clarithromycin; NS; 0.25g once a day | *Astragalus membranaceus* (Fisch.) Bge. *var. mongholicus* (Bge.) Hsiao (huang qi), *Ligusticum chuanxiong* Hort (chuan xiong), *Pogostemon cablin* (Blanco) Benth (huo xiang), *Scutellaria baicalensis* Georgi (huang qin), *Artemisia scoparia* Waldst. et Kit. (yin chen), *Lonicera japonica* Thunb (jin yin hua), *Gleditsia sinensis* Lam (zao ci), *Houttuynia cordata* Thunb (yu xing cao), *Patrinia villosa* (Thunb.) Juss (bai jiang cao), *Magnolia biondii* Pamp (xin yi), *Xanthium sibiricum* Patr (cang er zi), *Gardenia jasminoides* Elli (bai zhi), *Schizonepeta tenuifolia* Briq (jing jie), *Platycodon grandiflorum* (Jacq.) A. DC (jie geng), *Glycyrrhiza uralensis* Fisch (gan cao) |
| Xiong J 2016 (6) | *Long dan tong qiao wan* (龙胆通窍丸) LDTQW, granules; prepared on site at Hunan University of Chinese Medicine Affiliated Yueyang Hospital, Hunan China (湖南中医药大学附属岳阳医院制剂室生产, 60 g/瓶); 6g per dose, 3 times daily | oral clarithromycin; Jiangsu Hengrui Medicine and Pharmaceutical company, China (江苏恒瑞医药股份有限公司生产, 0.5 g/粒); 0.5g twice daily plus a nasal spray fluticasone; Glaxo Wellcome, S.A. State medical permit no: H20140117; 1 spray each side twice daily | *Gentiana manshurica* Kitage (long dan cao), *Pogostemon cablin* (Blanco) Benth (huo xiang), *Lonicera japonica* Thunb (jin yin hua), *Morus alba* L (sang bai pi), *Scutellaria baicalensis* Georgi (huang qin), *Gardenia jasminoides* Elli (zhi zi), *Magnolia biondii* Pamp (xin yi), *Ligusticum chuanxiong* Hort (chuan xiong), *Angelica dahurica* (Fisch. ex Hoffm.) Benth. et Hook. f (bai zhi), *Gleditsia sinensis* Lam (zao jiao ci), *Glycyrrhiza uralensis* Fisch (gan cao) |
| Yang L 2010 (NA) | No name; nasal steam inhalation, prepared on site at Guanganmen hospital Affiliated Hospital of China academy of Chinese medical sciences (中国中医科学院广安门医院), Beijing, China; nasal steam for 10 minutes, once a day | nasal steam inhalation of distilled water; prepared on site at Guanganmen hospital Affiliated Hospital of China academy of Chinese medical sciences, Beijing, China; nasal steam for 10 minutes, once a day | *Chrysanthemum morifolium* Ramat (ju hua), *Mentha haplocalyx* Briq (bo he), *Houttuynia cordata* Thunb (yu xing cao), *Magnolia biondii* Pamp (xin yi) |
| Zhang LY 2015 (7) | *Bi yuan tang* (鼻渊汤) BYT2, decoction; cooked by patients themselves; one packet per day, taken as two doses (morning and evening) | oral clarithromycin tablets; NS; 500mg, 1-2 tablets, 2-3 times a day plus triamcinolone acetonide nasal spray; NS; 1-3 sprays daily | *Xanthium sibiricum* Patr (cang er zi), *Magnolia biondii* Pamp (xin yi*), Saposhnikovia divaricata* (Turcz.) Schischk (fang feng), *Angelica dahurica* (Fisch. ex Hoffm.) Benth. et Hook. f (bai zhi), *Lonicera japonica* Thunb (jin yin hua), *Rubia cordifolia* L (qian cao), *Chrysanthemum indicum* L (ye ju hua) |
| Zhang XQ 2015 (NA) | *Tong bi tang* (通鼻汤) TBT, decoction; patients cook by themselves; 1 packet a day taken in 2 doses | oral penicillin; NS; 0.5g 3 times daily plus metronidazole; NS; 0.4g twice daily plus nasal drops (fu ma ye) containing 1% ephedrine twice daily | *Atractylodes macrocephala* Koidz (bai zhu), *Angelica dahurica* (Fisch. ex Hoffm.) Benth. et Hook. f (bai zhi), *Citrus reticulata* Blanco (chen pi), *Xanthium sibiricum* Patr (cang er zi), *Codonopsis pilosula* (Franch.) Nannf (dang shen), *Magnolia biondii* Pamp (xin yi), *Dioscorea opposita* Thunb (shan yao), *Poria cocos* (Schw.) Wolf (fu ling), *Ephedra sinica* Stapf (ma huang), *Mentha haplocalyx* Briq (bo he), *Centipeda minima* (L.) A. Br. et Aschers (e bu shi cao) |
| Zhang YF 2015 (NA) | *Xiang ju jiao nang* (香菊胶囊) XJJN, granules; Buchang pharma, Shandong, China. State medical permit no: Z19991040 (山东步长制药股份有限公司, 国药准字Z19991040); 0.9g per dose, 3 times daily | oral clarithromycin; Jiangsu Hengrui Medicine and Pharmaceutical company, China. State medical permit no: H20031041 (江苏恒瑞医药股份有限公司, 国药准字: H20031041); 0.5g once a day for 2 weeks, then 0.25g once a day for the rest of the study plus budesonide nasal spray; AstraZeneca AB. State medical permit no: J20090079) 1 spray per side twice daily plus oral eucalyptol, limonene and pinene capsules (Myrtol^®^); R.P.  Scherer GmbH Co.KG, State medical permit no: Z0100009; 0.3g, 3 times daily | *Platycarya strobilacea* Sieb.et Zucc (hua xiang shu guo xu), *Prunella vulgaris* L (xia ku cao), *Chrysanthemum indicum* L (ye ju hua), *Astragalus membranaceus* (Fisch.) Bge. *var. mongholicus* (Bge.) Hsiao (huang qi), *Magnolia biondii* Pamp (xin yi), *Saposhnikovia divaricata* (Turcz.) Schischk (fang feng), *Angelica dahurica* (Fisch. ex Hoffm.) Benth. et Hook. f (bai zhi) |
| Zhong MR 2020 (NA) | *Huang qin hua shi tang* (黄芩滑石汤) HQHST, decoction; NS; one bag per day, 250ml per dose, twice daily | oral cefuroxime; Zhejiang Jingxin Pharmaceutical Co. Ltd. China. Lot no: 20170123 (浙江京新药业股份有限公司, 批号: 20170123); 250mg per dose, twice daily | *Scutellaria baicalensis* Georgi (huang qin), Hydrated magnesium silicate (hua shi), *Poria cocos* (Schw.) Wolf (fu ling), *Areca catechu* L. (da fu pi), *Amomum krarvanh* Pierre ex Gagnep (bai kou ren), *Tetrapanax papyrifera* (Hook.) K. Koch (tong cao), *Polyporus umbellatus* (Pers.) Fires (zhu ling), *Xanthium sibiricum* Patr (cang er zi), *Angelica dahurica* (Fisch. ex Hoffm.) Benth. et Hook. f (bai zhi), *Platycodon grandiflorum* (Jacq.) A. DC (ji geng), *Pueraria lobata* (Willd.) Ohwi (ge gen), *Glycyrrhiza uralensis* Fisch (gan cao) |
| Zhou L 2013 (8) | *Long dan xie gan tang* (龙胆泻肝汤) LDXGT, decoction; NS; one packet per day, 200ml per dose taken 30-60min after meal | oral placebo; NS; 200ml per dose taken 30-60min after meal | *Gentiana manshurica* Kitage (long dan cao), *Scutellaria baicalensis* Georgi (huang qin), *Gardenia jasminoides* Elli (zhi zi), *Alisma orientalis* (Sam.) Juzep (ze xie), *Clematis armandii* Franch (chuan mu tong), *Plantago asiatica* L (che qian zi), *Angelica sinensis* (Oliv.) Diel (dang gui), *Rehmannia glutinosa* Libosch (di huang), *Bupleurum chinense* DC (chai hu), *Glycyrrhiza uralensis* Fisch (gan cao) |
| Zhu XP 2017 (NA) | *Xing qiao tang* (醒窍汤) XQT, decoction; patients cook by themselves; 1 packet per day, taken as two doses (morning and evening) | oral amoxicillin; Shandong Lunan Beite Pharmaceutical Co., Ltd. China. State medicine permit no: H20050586 (山东鲁南贝特制药股份有限公司, 批号 H20050586); 685.5mg, twice daily plus budesonide nasal spray; Jianqiao Xinyuan Pharmaceutical Technology Co., Ltd. Taiwan (台湾健乔信元医药生物股份有限公司*,* 批号 HC20130020); 1 spray twice daily plus a nasal spray containing sea water; Shenyang Dade medical devices Co., Ltd. (沈阳大得医疗器械产品有限公司); 5 times a day | *Pogostemon cablin* (Blanco) Benth (huo xiang), *Scutellaria baicalensis* Georgi (huang qin), *Houttuynia cordata* Thunb (yu xing cao), *Xanthium sibiricum* Patr (cang er zi), *Magnolia biondii* Pamp (xin yi), *Angelica dahurica* (Fisch. ex Hoffm.) Benth. et Hook. f (bai zhi), *Platycodon grandiflorum* (Jacq.) A. DC (jie geng), *Glycyrrhiza uralensis* Fisch (gan cao) |

Abbreviations: CHM: Chinese herbal medicine; NA: not applicable; NS: not specified.

Note: 1) Chinese and scientific names are based on: Chinese Pharmacopoeia Commission. (2015). *Pharmacopoeia of the People's Republic of China* [Zhonghua Renmin Gonghe Guoyaodian]. Beijing: China Medical Science Press (Chinese Edition)

**Funding sources**:

1. 基金项目: 湖北省十堰市科技局课题: EK2012D150022000383;
2. 基金项目:珠海市科技局基金: 2012D0401990021);
3. 基金项目: 国家自然科学基金: 81373699; 中医诊断学国家重点学科开放基金: 2014-15; 湖南省教育厅: 14C0875; 湖南省教育厅一般项目 除外: 226; 湖南中医药大学学位与研究生教育教学改革项目: 2013JG03);
4. 基金项目: 广东省中医药局资助项目: 2009294);
5. 基金项目: 国家自然科学基金 (编号: 81960890) 及云南省科技厅中医联合专项课题基金资助 (编号: 2018FF001- 034)
6. 基金项目：湖南省科技厅科技计划项目: 2013FJ3021; 湖南省中 医药管理局科技项目: 2012105);
7. 河北省卫生和计划生育委员会医学科学研究重点课题支持项目: 20120464);
8. 基金项目: 四川省教育厅项目资助: 11ZB183).

**S4 Table. Main ingredients of the Chinese herbal medicines**

| **Scientific name (name in Chinese pin yin)^1^** | **N CHMs^2^** |
| --- | --- |
| *Magnolia biondii* Pamp. (xin yi 辛夷) | 30 |
| *Angelica dahurica* (Fisch. ex Hoffm.) Benth. et Hook. f (bai zhi 白芷) | 29 |
| *Xanthium sibiricum* Patr. (cang er zi 苍耳子) | 26 |
| *Scutellaria baicalensis* Georgi (huang qin 黄芩) | 22 |
| *Glycyrrhiza uralensis* Fisch. (gan cao 甘草) | 16 |
| *Mentha haplocalyx* Briq. (bo he 薄荷) | 16 |
| *Astragalus membranaceus* (Fisch.) Bge. var. *mongholicus* (Bge.) Hsiao (huang qi 黄芪) | 14 |
| *Ligusticum chuanxiong* Hort. (chuan xiong 川芎) | 12 |
| *Platycodon grandiflorum* (Jacq.) A. DC. (jie geng 桔梗) | 11 |
| *Poria cocos* (Schw.) Wolf (fu ling 茯苓) | 10 |

Abbreviations: CHM: Chinese herbal medicine; N: number.

Notes: 1) Chinese and scientific names are based on: Chinese Pharmacopoeia Commission. (2015). *Pharmacopoeia of the People's Republic of China* [Zhonghua Renmin Gonghe Guoyaodian]. Beijing: China Medical Science Press (Chinese Edition); 2) Based on formulae in 36 test groups, excluding repeated formulae in same study.

**S5 Table. Risk of bias assessments for included studies**

| **Ref No** | **Included studies** | **Risk of Bias Categories** | | | | | | |
| --- | --- | --- | --- | --- | --- | --- | --- | --- |
|  | **Study name** | **SG** | **AC** | **BPt** | **BPn** | **BOA** | **IOD** | **SOR** |
| 41 | Chen TT 2017 | U | U | H | H | H | L | U |
| 42 | Chu XY 2017 | U | U | H | H | H | L | U |
| 43 | Dai RZ 2015 | U | U | H | H | H | L | U |
| 44 | Deng QH 2016 | U | U | H | H | H | L | U |
| 45 | Du JW 2016 | L | U | H | H | H | L | U |
| 58 | Guo L 2015 | L | U | H | H | H | L | U |
| 46 | Hong HY 2015 | U | U | H | H | H | L | U |
| 47 | Hu FL 2015 | L | U | H | H | H | L | U |
| 35 | Huang JY 2017 | U | U | H | H | H | L | U |
| 31 | Jiang RS 2012 | L | L | L | L | L | U^1^ | U |
| 34 | Li MJ 2014 | L | U | H | H | H | L^2^ | U |
| 33 | Lin L 2015 | U | U | H | H | H | L | U |
| 48 | Liu HL 2017 | L | U | H | H | H | L | U |
| 39 | Liu JB 2011 | L | L | L | L | L | L^3^ | U |
| 49 | Liu Q 2015 | L | U | H | H | H | L | U |
| 50 | Wang C 2014 | L | U | H | H | H | L | U |
| 51 | Wang G 2013 | L | U | H | H | H | L | U |
| 52 | Wang KQ 2016 | L | U | H | H | H | L | U |
| 53 | Wang P 2015 | U | U | H | H | H | U^4^ | U |
| 40 | Xiong J 2016 | L | U | H | H | H | L | U |
| 37 | Yang L 2010 | L | L | L | L | L | L | U |
| 54 | Zhang LY 2015 | U | U | H | H | H | L | U |
| 55 | Zhang XQ 2015 | L | U | H | H | H | L | U |
| 56 | Zhang YF 2015 | U | U | H | H | H | L | U |
| 38 | Zhou L 2013 | L | U | L | U | U | L | U |
| 57 | Zhu XP 2017 | U | U | H | H | H | L | U |
| 59 | Cai CJ 2019 | L | U | H | H | H | L | H |
| 60 | Liao WT 2020 | L | U | H | H | H | L | U |
| 61 | Wang J 2020 | L | U | U | H | U | L | U |
| 36 | Zhong MR 2020 | L | U | H | H | H | L | U |
| 32 | Lin L 2020 | L | U | L | U | U | U | U |
| 62 | Wang H 2009 | U | U | H | H | U | L | U |
| 63 | Qiang JH 2011 | U | U | H | H | U | L | U |
| 64 | Wu MM 2022 | U | U | H | H | U | L | U |
|  | Number ‘Low’ (%) | 20 L (58.8) | 3 L (8.8) | 5 L (14.7) | 3 L (8.8) | 3 L (8.8) | 30 L (88.2) | 0 L (0) |

Abbreviations: AC: allocation concealment; BOA: blinding of outcome assessment; BPn: blinding of personnel; BPt: blinding of participants; H: high risk; IOD: incomplete outcome data; L: low risk; SG: sequence generation; SOR: selective outcome reporting; U: unclear risk or no information specified.

Notes: 1) Jiang RS 2012: IOD U over 20% dropouts (n=30), no reasons given, no intent to treat analysis, but completers were balanced between groups; 2) Li MJ 2014: IOD L had 4 dropouts/exclusions in the test group and 7 in the control (total 10.2%), no intent to treat analysis; 3) Liu JB 2011: IOD L had 4 dropouts in the test group (total 8.3%) but used intent to treat analysis; 4) Wang P 2015: IOD U over 30% dropouts, no reasons given, no intent to treat analysis.

**S6 Table. SNOT: Meta-analysis results for chronic rhinosinusitis at end of treatment and changes within treatment and control groups**

| **Comparison** | **N. studies (N participants at EoT: T, C) Duration [study name]** | **T vs C at EoT; MD [95% CI] *I^2^*** | **T group change (baseline vs EoT); MD [95% CI] *I^2^*** | **C group change (baseline vs EoT); MD [95% CI] *I*^2^** |
| --- | --- | --- | --- | --- |
| **Oral CHM vs inactive control** | | | | |
| Oral LDXGT vs placebo^1^ (SNOT-20) | 1 (30,30) 2wks [Zhou L 2013] BI, T higher | -4.90 [-8.12, -1.68]* | -12.80 [-15.43, -10.17]* | -4.20 [-7.37, -1.03]* |
| Oral LHQW vs placebo (SNOT-22) | 1 (70/70) 30days [Lin L 2020] | T median 46.0, range 35.0–56.0; C median 55.5, range 48–68^2^ | Sig reduced | No sig change |
| **Oral CHM plus placebo vs pharmacotherapy (PT) plus placebo^3^** | | | | |
| Oral CEZS (modified) vs erythromycin (double blind) | 1 (26,27) 8wks [Jiang RS 2012] | -0.40 [-1.94, 1.14] | -6.90 [-8.65, -5.15]* | -5.70 [-7.28, -4.12]* |
| **Oral CHM plus pharmacotherapy vs same PT (IM)^3^** | | | | |
| Oral LDXGT plus clarithromycin plus nasal mometasone furoate spray vs same PT | 1 (30,30) 12wks [Chen TT 2017] | -2.93 [-5.11, -0.75]*  5 items -1.03 [-1.68, -0.38]* | -14.93 [-18.49, -11.37]*  5 items -6.33 [-7.35, -5.31]* | -11.60 [-16.07, -7.13]*  5 items -6.00 [-7.06, -4.94]* |
| Oral BYTQKL plus clarithromycin vs same PT | 1 (32,32) 4wks [Hong HY 2015] | -4.30 [-7.25, -1.35]* | -7.20 [-10.45, -3.95]* | -2.72 [-5.91, 0.47] |
| Oral GLXDD plus mometasone furoate nasal spray vs same PT | 1 (28.27) 4wks [Cai CJ 2019] | -3.75 [-5.83, -1.67]* | -15.65 [-18.93, -12.37]* | -10.61 [-14.41, -6.81]* |
| **Pool**: Oral CHM plus PT vs PT EoT | 3 (90,89) 4-12wks [Chen TT 2017, Hong HY 2015, Cai CJ 2019] | -3.55 [-4.89, -2.21]* 0% | -12.57 [-17.98, -7.16]* 87% | -8.17 [-14.04, -2.30]* 86% |
| **Nasal CHM plus pharmacotherapy vs same PT (IM)^4^** | | | | |
| XZDBY nasal drops plus oral roxithromycin plus fluticasone nasal spray | 1 (342,256) 16wks [30 Wang P 2015] BI, T lower | -6.00 [-7.66, -4.34]* | -18.94 [-20.59, -17.29]* | -15.16 [-16.94, -13.38]* |
| **Oral plus nasal CHM plus pharmacotherapy vs same PT (IM)^3^** | | | | |
| BYKT nasal wash plus Oral BYKT plus oral amoxicillin plus budesonide nasal spray plus sea water nasal spray vs same PT | 1 (60,60) 12wks [Guo L 2015] | -6.42 [-7.41, -5.43]* | -30.68 [-32.41, -28.95]* | -23.68 [-25.58, -21.78]* |

* significant difference.

Abbreviations: C: control group; CHM: Chinese herbal medicine; CI: confidence interval; CRS: chronic rhinosinusitis; EoT: end of treatment; *I*^2^: index of heterogeneity; IM: integrative medicine; MD: mean difference; Mod: modified; N: number; PT: pharmacotherapies; SNOT: Sino-Nasal Outcome Test (20 or 22 in one study only); T: treatment group; vs: versus; wks: weeks.

Notes: 1) at baseline the test group of this study (Zhou L 2013) had higher SNOT-20 scores than the placebo group (MD 3.70 [1.14, 6.26]); 2) the authors reported the scores in the CHM group were significantly lower; 3) baseline scores showed no significant difference between groups for each study in this comparison; 4) at baseline, there was a significant difference between groups (MD –2.22 [–3.98, –0.46]) in favour of the IM group.

**S7 Table. SNOT-20 subscales Meta-analysis results for CRS at end of treatment change within treatment groups and control groups**

| **Comparison (IM)** | **N. studies (N. participants at EoT: T, C) Duration [study name]** | **T vs C at EoT; MD [95% CI] *I^2^*** | **T group change (baseline vs EoT); MD [95% CI] *I^2^*** | **C group change (baseline vs EoT); MD [95% CI] *I*^2^** |
| --- | --- | --- | --- | --- |
| Oral TBT plus PT vs PT: penicillin plus metronidazole plus nasal drops containing 1% ephedrine^1^ | 1 (45,45) 10d [Zhang XQ 2015] No BI | NSx: -4.58 [-5.07, -4.09]* | NSx: -10.91 [-11.36, -10.46]* | NSx: -6.60 [-7.13, -6.07]* |
|  |  | RSx: -2.18 [-2.63, -1.73]* | RSx: -5.75 [-6.16, -5.34]* | RSx: -3.52 [-3.99, -3.05]* |
|  |  | SF: -1.48 [-1.99, -0.97]* | SF: -6.49 [-6.97, -6.01]* | SF: -4.96 [-5.57, -4.35]* |
|  |  | PF: -3.62 [-4.19, -3.05]* | PF: -11.31 [-11.87, -10.75]* | PF: -7.66 [-8.27, -7.05]* |

* significant difference.

Abbreviations: C: control group; CI: confidence interval; d: days; CRS: chronic rhinosinusitis; EoT: end of treatment; RSx: related symptoms; *I*^2^: index of heterogeneity; IM: integrative medicine; MD: mean difference; N: number; PF: psychological function; PT: pharmacotherapies; NSx: nasal symptoms; SF: sleep function; SNOT-20-subscales: Sino-Nasal Outcome Test (SNOT)-20 subscales; T: test group; vs: versus; wks: weeks.

Note: 1) baseline scores showed no significant difference between groups for all studies in this comparison.

**S8 Table. VAS-TNS Meta-analysis results for CRS at end of treatment** **and change within treatment and control groups**

| **Comparison** | **N. studies (N. participants at EoT: T, C) Duration [study name]** | **T vs C at EoT; MD [95% CI] *I^2^*** | **T group change (baseline vs EoT); MD [95% CI] *I^2^*** | **C group change (baseline vs EoT); MD [95% CI] *I*^2^** |
| --- | --- | --- | --- | --- |
| **Oral CHM vs inactive controls** | | | | |
| Oral LHQW vs placebo | 1 (70 /70) 30d [Lin L 2020] | T median 4.5, range 3.0–5.5; C median 5.5, range 5.0–8.0 | Sig reduced | No sig change |
| Oral LDXGT vs placebo ^1^ | 1 (30,30) 2wks [Zhou L 2013] BI, T lower | -1.40 [-1.53, -1.27]* | -2.10 [-2.29, -1.91]* | -1.00 [-1.15, -0.85]* |
| **Oral CHM vs pharmacotherapy (PT)^2^** | | | | |
| Oral BYSJN vs clarithromycin (double blind)^2^ | 1 (24,24) 12wks [Liu JB 2011] | 0.88 [-0.08, 1.84] | -3.00 [-3.75, -2.25]* | -3.92 [-4.82, -3.02]* |
| Oral LDTQW vs clarithromycin plus fluticasone nasal spray (open label)^2^ | 1 (40,40) 4wks [Xiong J 2016] | -0.08 [-0.75, 0.59] | -3.53 [-4.26, -2.80]* | -3.47 [-4.13, -2.81]* |
| Oral TBXTKL vs clarithromycin (open label) ^3^ | 1 (35,34) 4wks [Wu MM 2022] | -2.06 [-4.65, 0.53] | -5.54 [-7.77, -3.31]* | -3.54 [-5.98, -1.10]* |
| **Pool:** all studies (MD/SMD)^3^ | 3 (99,98) 4-12 wks [Liu JB 2011, Xiong J 2016, Wu MM 2022] | MD 0.03 [-1.06, 1.12] 64% p=0.06  SMD 0.00 [-0.46, 0.47] 63% | MD -3.56 [-4.46, -2.66]* 58%;  SMD -1.79 [-2.49, -1.10]* 76% | MD -3.62 [-4.14, -3.10] 0%;  SMD -1.78 [-2.96, -0.60]* 91% |
| **Sensitivity:** studies with comparable VAS results as MD | 2 (64,64) 4-12 wks [Liu JB 2011, Xiong J 2016] | 0.34 [-0.60, 1.27] 61% p=0.11 | -3.27 [-3.80, -2.75]* 0% | -3.63 [-4.16, -3.10]* 0% |
| **Oral CHM plus pharmacotherapy vs the same PT (IM)** | | | | |
| Oral BYSW plus expectorant vs same PT^2,5^ | 1 (30,30) 4wks [Liu HL 2017a CHMa] no BI | -1.64 [-2.17, -1.11]* | -5.14 [-5.76, -4.52]* | -3.80 [-4.44, -3.16]* |
| Oral TQBYKL plus expectorant vs same PT^2,5^ | 1 (30,30) 4wks [Liu HL 2017b CHMb] no BI | -0.40 [-1.22, 0.42] | -4.06 [-4.77, -3.35]* | As above |
| Oral BDYKFY plus clarithromycin vs same PT^2^ | 1 (43,43) 12wks [Liu Q 2015] no BI | -1.07 [-1.28, -0.86]* | -5.38 [-5.84, -4.92]* | -4.25 [-4.77, -3.73]* |
| Oral BYSKFY plus clarithromycin plus eucalyptol limonene and pinene capsules plus nasal wash of saline vs same PT^2^ | 1 (48,48) 12wks [Wang C 2014] no BI | -2.03 [-2.56, -1.50]* | -4.03 [-4.99, -3.07]* | -2.20 [-3.13, -1.27]* |
| Oral BYT plus clarithromycin plus triamcinolone nasal spray vs same PT^2^ | 1 (40,40) 3wks [Zhang LY 2015] no BI | -2.50 [-2.88, -2.12]* | -7.20 [-7.68, -6.72]* | -4.41 [-4.84, -3.98]* |
| Oral TBT plus penicillin plus metronidazole plus nasal drops containing 1% ephedrine vs same PT^2^ | 1 (45,45) 10d EoT. 30d FU [Zhang XQ 2015] | EoT –1.19 [–1.38, –1.00]*  FU -0.93 [-1.11, -0.75]* | -5.23 [-5.40, -5.06]*  -6.30 [-6.45, -6.15]* | -4.00 [-4.19, -3.81]*  -5.33 [-5.52, -5.14]* |
| Oral XQT plus amoxicillin plus budesonide nasal spray plus sea water spray vs same PT^2^ | 1 (48,48) 28d [Zhu XP 2017] | -1.79 [-2.03, -1.55]* | -6.66 [-6.88, -6.44]* | -4.92 [-5.16, -4.68]* |
| **Pool:** oral CHM plus PT vs PT at EoT | 6 studies, 7 groups (284,254) 3-12wks [Liu HL 2017 CHM1, Liu HL 2017 CHM2, Liu Q 2015, Wang C 2014, Zhang LY 2015, Zhang XQ 2015, Zhu XP 2017] | –1.55 [–1.97, –1.13]* 91% | -5.45 [-6.21, -4.69]* 96% | -4.04 [-4.56, -3.52]* 91% |
| **Sensitivity:** oral CHM plus PT vs PT 12wk studies of oral clarithromycin | 2 (91,91) 12wks [Liu Q 2015, Wang C 2014] | -1.52 [-2.46, -0.58]* 91% | -4.77 [-6.09, -3.46]* 84% | -3.26 [-5.27, -1.26]* 93% |
| **Nasal CHM plus pharmacotherapy vs same PT (IM)** | | | | |
| XZDBY nasal drops plus oral roxithromycin plus fluticasone nasal spray vs same PT^5^ | 1 (342,256) 16wks [Wang P 2015] BI | -0.13 [-0.40, 0.14] | -4.16 [-4.44, -3.88]* | -3.68 [-3.98, -3.38]* |
| **Oral plus nasal CHM plus pharmacotherapy vs same PT (IM)^2^** | | | | |
| BYKT nasal wash plus oral BYKT plus oral amoxicillin plus budesonide nasal spray plus sea water nasal spray vs same PT^2^ | 1 (60,60) 12wks [Guo L 2015] | -0.73 [-1.11, -0.35]* | -4.89 [-5.45, -4.33]* | -4.07 [-4.62, -3.52]* |

* significant difference.

Abbreviations: C: control group; CHM: Chinese herbal medicine; CI: confidence interval; CRS: chronic rhinosinusitis; d: days; EoT: end of treatment; *I*^2^: index of heterogeneity; IM: integrative medicine; MD: mean difference; N: number; PT: pharmacotherapies; T: treatment group; VAS-TNS: Visual analogue scale scores for total nasal symptoms; vs: versus; wks: weeks.

Notes: 1) At baseline the test group of this study (Zhou L 2013) had lower VAS-TNS scores than the placebo group (MD -0.30 [-0.51, -0.09]); 2) baseline scores showed no significant difference between groups; 3) the scores for VAS-TNS in Wu MM 2022 were considerably greater thant in the other two studies, suggesting the scales may have been different, so an SMD analysis was added; 4) Since this study (Liu HL 2017a&b) included two test groups, when data were pooled the number in the control group was halved from 30 to 15 to avoid double-counting; 5) At baseline the test group of this study (Wang P 2015) had higher VAS-TNS scores than the control group (MD 0.35 [0.05, 0.65]).

**S9 Table. VAS-IS Meta-analysis results for CRS at end of treatment and change within treatment groups and control groups**

| **Comparison (all IM)** | **N. studies (N. participants at EoT: T, C) Duration [study name]** | **T vs C at EoT; MD [95% CI] *I^2^*** | **T group change (baseline vs EoT); MD [95% CI] *I^2^*** | **C group change (baseline vs EoT) MD [95% CI] *I*^2^** |
| --- | --- | --- | --- | --- |
| Nasal steam CHM decoction vs placebo^1^ | 1 (26,26) 3d [Yang L 2010], adults | NB: -1.54 [-2.10, -0.98]* | NB: -2.43 [-2.95, -1.91]* | NB: -0.65 [-1.17, -0.13]* |
| Oral BYTQKL plus oral clarithromycin vs same PT^1^ | 1 (32,32) 4wks [Hong HY 2015], adults, no BI | NB: -1.02 [-2.11, 0.07] | NB: -1.99 [-3.24, -0.74]* | NB: -0.90 [-2.07, 0.27] |
|  |  | ND: -1.15 [-2.02, -0.28]* | ND: -1.99 [-2.89, -1.09]* | ND: -0.95 [-2.02, 0.12] |
|  |  | PD: -0.02 [-1.08, 1.04] | PD: -0.71 [-1.75, 0.33] | PD: -0.64 [-2.01, 0.73] |
|  |  | HY: -0.47 [-1.12, 0.18] | HY: -0.91 [-1.70, -0.12]* | HY: -0.50 [-1.26, 0.26] |
| MYBYPWJ nasal spray plus oral cefixime or roxithromycin vs same PT^1^ | 1 (49,49) 20 days, 12wks (FU) [Wang G 2013], children, no BI | NB: -1.09 [-1.60, -0.58]* | NB: -3.33 [-3.80, -2.86]* | NB: -2.29 [-2.80, -1.78]* |
|  |  | ND: -1.03 [-1.55, -0.51]* | ND: -4.46 [-4.96, -3.96]* | ND: -3.40 [-3.99, -2.81]* |
|  |  | HY: -0.09 [-0.38, 0.20] | HY: -0.59 [-0.89, -0.29]* | HY: -0.53 [-0.84, -0.22]* |
|  |  | HD: -0.36 [-0.72, 0.00] | HD: -2.40 [-2.87, -1.93]* | HD: -1.96 [-2.48, -1.44]* |
| BYT nasal wash plus oral cefaclor plus budesonide nasal spray vs same PT^1^ | 1 (84,83) 12 wks [Wang KQ 2016], children, no BI | NB: -1.33 [-1.47, -1.19]* | NB: -2.78 [-2.98, -2.58]* | NB: -1.52 [-1.75, -1.29]* |
|  |  | ND: -1.41 [-1.65, -1.17]* | ND: -4.52 [-4.77, -4.27]* | ND: -3.08 [-3.38, -2.78]* |
|  |  | HD: -1.19 [-1.28, -1.10]* | HD: -2.63 [-2.75, -2.51]* | HD: -1.46 [-1.60, -1.32]* |

* significant difference.

Abbreviations: C: control group; CHM: Chinese herbal medicine; CI: confidence interval; d: days; CRS: chronic rhinosinusitis; EoT: end of treatment; FU: follow-up; HD: headache; HY: hyposmia; *I*^2^: index of heterogeneity; IM: integrative medicine; MD: mean difference; N: number; NB: nasal blockage; ND: nasal discharge; PD: post-nasal drip; PT: pharmocotherapies; T: test group; VAS-IS: Visual analog scale scores for individual symptoms; vs: versus; wks: weeks.

Note: 1) baseline scores showed no significant difference between groups for each study in this comparison.

**S10 Table. LM Meta-analysis results for CRS at end of treatment and change within treatment and control groups**

| **Comparison** | **N. studies (N. participants at EoT: T, C) Duration [study name]** | **T vs C at EoT; MD [95% CI] *I^2^*** | **T group change (baseline vs EoT); MD [95% CI] *I^2^*** | **C group change (baseline vs EoT); MD [95% CI] *I*^2^** |
| --- | --- | --- | --- | --- |
| **Nasal CHM vs nasal saline^1^** | | | | |
| Nasal YJPDHJ vs nasal 0.9% saline | 1 (30,31) 2wks [Wang J 2020] | -2.45 [-4.59, -0.31]* | -5.10 [-7.46, -2.74]* | -3.00 [-5.04, -0.96]* |
| **Oral CHM vs pharmacotherapy (PT)** | | | | |
| Oral CEZS (modified) vs oral erythromycin (double blind)^2^ | 1 (26,27) 8wks [Jiang RS 2012] BI, T lower | -0.10 [-0.24, 0.04] | -0.40 [-0.56, -0.24]* | -0.50 [-0.61, -0.39]* |
| Oral BYSJN vs oral clarithromycin (double blind)^1^ | 1 (24,24) 12wks [Liu JB 2011] | 1.93 [0.51, 3.35]* | -3.24 [-4.68, -1.80]* | -5.13 [-6.63, -3.63]* |
| Oral TBXTKL vs clarithromycin | 1 (35,34) 4wks [Wu MM 2022] | -0.48 [-1.67, 0.71] | -0.68 [-1.95, 0.59] | -0.27 [-1.56, 1.02] |
| **Pool**: Oral CHM vs oral antibiotics | 3 (85,85) 4-12wks [Jiang RS 2012, Liu JB 2011, Wu MM 2022] | 0.31 [-0.78, 1.40] 76% | -1.32 [-2.86, 0.22] 87% | -1.89 [-4.33, 0.55] 97% |
| **Sensitivity 1:** Oral CHM vs oral antibiotics (double blind) | 2 (50,51) 8-12wks [Jiang RS 2012, Liu JB 2011] | 0.79 [-1.19, 2.76] 87% | -1.73 [-4.50, 1.05]* 93% | -2.75 [-7.29, 1.78]* 97% |
| **Sensitivity 2:** Oral CHM vs oral antibiotics (no BI) | 2 (58,58) 4-12wks [Liu JB 2011, Wu MM 2022] | -2.68 [-7.45, 2.08] 96% | -1.94 [-4.45, 0.57] 85% | -2.68 [-7.45, 2.08] 96% |
| **Oral CHM plus pharmacotherapy vs the same PT (IM)^1^** | | | | |
| Oral LDXGT plus oral clarithromycin plus mometasone furoate nasal spray vs same PT | 1 (30,30) 12wks [Chen TT 2017] | -1.27 [-2.38, -0.16]* | -7.47 [-9.17, -5.77]* | -4.76 [-6.29, -3.23]* |
| Oral BYSKFY plus oral clarithromycin vs same PT | 1 (42,42); 12wks; [Hu FL 2015] | -1.60 [-1.84, -1.36]* | -3.90 [-4.39, -3.41]* | -2.40 [-2.91, -1.89]* |
| Oral BDYKFY plus oral clarithromycin vs same PT | 1 (43,43) 12wks [Liu Q 2015] | -1.07 [-1.28, -0.86]* | -5.78 [-6.24, -5.32]* | -4.65 [-5.17, -4.13]* |
| Oral BYSKFY plus oral clarithromycin plus eucalyptol limonene and pinene capsules plus saline nasal wash vs same PT | 1 (48,48) 12wks [Wang C 2014] | -2.00 [-2.33, -1.67]* | -4.33 [-4.96, -3.70]* | -2.35 [-3.00, -1.70]* |
| Oral XJJN plus oral clarithromycin plus eucalyptol limonene and pinene capsules plus nasal budesonide vs same PT | 1 (69,65) 12 wks, 24wks FU; [Zhang YF 2015] | -2.70 [-3.21, -2.19]* | -7.14 [-7.90, -6.38]* | -4.86 [-5.65, -4.07]* |
| **Pool**: Oral CHM plus PT vs same PT at EoT | 4 (163,163) 12wks [Chen TT 2017, Hu FL 2015, Liu Q 2015, Wang C 2014] | -1.51 [-1.98, -1.04]* 88% | -5.18 [-6.38, -3.99]* 93% | -3.47 [-4.83, -2.11]* 94% |
| **Sensitivity**: T group same CHM (BYSKFY) at 12wks EoT | 2 (90,90) 12wks [Hu FL 2015, Wang C 2014] | -1.78 [-2.17, -1.39]* 73% | -4.07 [-4.48, -3.66]* 11% | -2.38 [-2.78, -1.98]* 0% |

* significant difference

Abbreviations: BI: baseline imbalance; C: control group; CHM: Chinese herbal medicine; CI: confidence interval; CRS: chronic rhinosinusitis; EoT: end of treatment; FU: follow-up; *I*^2^: index of heterogeneity; IM: integrative medicine; MD: mean difference; LM: Lund-Mackay computed tomography score; N: number; PT: pharmacotherapies; T: treatment group; vs: versus; wks: weeks.

Notes: 1) baseline scores showed no significant difference between groups for this comparison; 2) baseline scores showed a significant difference between groups (MD -0.20 [-0.34, -0.06]) indicating the CHM group was worse.

**S11 Table. LK Meta-analysis results for CRS at end of treatment and change within treatment and control groups**

| **Comparison** | **N. studies (N. participants at EoT: T, C) Duration [study name]** | **T vs C at EoT; MD [95% CI] *I^2^*** | **T group change (baseline vs EoT); MD [95% CI] *I^2^*** | **C group change (baseline vs EoT); MD [95% CI] *I*^2^** |
| --- | --- | --- | --- | --- |
| **Nasal CHM vs nasal saline^1^** | | | | |
| Nasal YJPDHJ vs nasal 0.9% saline | 1 (30,31) 2wks [Wang J 2020] | -0.65 [-1.27, -0.03]* | -2.27 [-2.85, -1.69]* | -1.97 [-2.59, -1.35]* |
| **Oral CHM vs pharmacotherapy (PT) ^1^** | | | | |
| Oral LDTQW vs oral clarithromycin plus fluticasone nasal spray | 1 (40,40) 4wks [Xiong J 2016] | -0.73 [-1.21, -0.25]* | -4.39 [-4.92, -3.86]* | -4.05 [-4.41, -3.69]* |
| Oral TBXTKL vs clarithromycin | 1 (35,34) 4wks [Wu MM 2022] | -0.69 [-1.23, -0.15]* | -1.00 [-1.65, -0.35]* | -0.41 [-1.01, 0.19] |
| **Pool**: Oral CHM plus PT vs same PT (EoT) | 2 (75,74) 4wks [Xiong J 2016, Wu MM 2022] | -0.71 [-1.07, -0.35]* 0% | -2.70 [-6.02, 0.62] 98% | -2.24 [-5.81, 1.33] 99% |
| **Oral CHM plus pharmacotherapy vs the same PT (IM)^1^** | | | | |
| Oral GLXDD plus mometasone furoate nasal spray vs same PT | 1 (28.27) 4wks [Cai CJ 2019] | -0.42 [-0.80, -0.04]* | -3.93 [-4.47, -3.39]* | -3.43 [-3.94, -2.92]* |
| Oral LDXGT plus oral clarithromycin plus mometasone furoate nasal spray vs same PT | 1 (30,30) 12wks [Chen TT 2017] | -0.80 [-1.32, -0.28]* | -3.60 [-4.52, -2.68]* | -2.17 [-3.08, -1.26]* |
| Oral BYSKFY plus oral clarithromycin vs same PT | 1 (58,58) 15d [Dai RZ 2015] | -2.07 [-2.85, -1.29]* | -9.01 [-9.80, -8.22]* | -6.55 [-7.35, -5.75]* |
| Oral BDYKFY plus oral clarithromycin vs same PT | 1 (44,45) 20d [Du JW 2016] | -2.49 [-3.35, -1.63]* | -9.38 [-10.45, -8.31]* | -6.35 [-7.54, -5.16]* |
| Oral BYTQKL plus oral clarithromycin vs same PT | 1 (32,32) 4wks [Hong HY 2015] | -1.09 [-1.51, -0.67]* | -2.69 [-3.20, -2.18]* | -1.69 [-2.28, -1.10]* |
| Oral BDYKFY plus oral clarithromycin vs same PT | 1 (43,43) 12wks [Liu Q 2015] | -1.10 [-1.20, -1.00]* | -3.03 [-3.20, -2.86]* | -1.74 [-1.91, -1.57]* |
| Oral XJJN plus oral clarithromycin plus nasal budesonide plus eucalyptol, limonene, pinene capsules vs same PT | 1 (69,65); 12wks [Zhang YF 2015] | EoT: -0.08 [-0.67, 0.51];  24wks FU: -0.67 [-1.08, -0.26]* | EoT: -3.45 [-4.05, -2.85]*;  24wks FU: -5.57 [-6.06, -5.08]* | EoT: -2.98 [-3.63, -2.33]*;  24wks FU: -4.51 [-5.11, -3.91]* |
| **Pool**: Oral CHM plus PT vs same PT (EoT) | 7 (304,300); 15d-12 wks; [Cai CJ 2019, Chen TT 2017, Dai RZ 2015, Du JW 2016. Hong HY 2015, Liu Q 2015, Zhang YF 2015] | -1.06 [-1.46, -0.65]* 85% | -4.97 [-6.45, -3.49]* 98% | -3.51 [-4.75, -2.27]* 97% |
| **Sensitivity 1:** studies using CHM plus oral clarithromycin only (at EoT) | 4 studies (177, 178) 15 days-12 weeks [Dai RZ 2015, Du JW 2016, Hong HY 2015, Liu Q 2015] | -1.53 [-2.05, -1.02]* 81% | -5.99 [-8.75, -3.23]* 99% | -4.03 [-6.28, -1.79]* 98% |
| **Sensitivity 2:** studies of same duration (12wks, EoT) | 3 studies (142, 138) [Chen TT 2017, Liu Q 2015, Zhang YF 2015] | -0.72 [-1.29, -0.14]* 84% | -3.19 [-3.52, -2.85]* 34% | -2.26 [-3.10, -1.42]* 85% |
| **Sensitivity 3:** studies using BDYKFY (EoT) | 2 studies (87, 88) 20 days-12 weeks [Liu Q 2015, Du JW 2016] | -1.73 [-3.08, -0.37]* 90% | -6.18 [-12.40, 0.04]* 99% | -4.01 [-8.52, 0.51]* 98% |
| **Nasal CHM plus pharmacotherapy vs same PT (IM)^1^** | | | | |
| MYBYPWJ nasal spray plus oral cefixime or roxithromycin vs same PT | 1 (49,49); 20 days, 6wks (FU), 12wks (FU); [Wang G 2013] | 6wks FU: -0.56 [-1.05, -0.07]*;  12wks FU: -1.23 [-1.61, -0.85]* | 6wks FU: -2.57 [-3.05, -2.09]*;  12wks FU: -3.48 [-3.92, -3.04]* | 6wks FU: -2.14 [-2.68, -1.60]*;  12wks FU: -2.38 [-2.87, -1.89]* |
| **Oral plus nasal CHM plus pharmacotherapy vs same PT (IM) ^1^** | | | | |
| BYKT nasal wash plus Oral BYKT plus oral amoxicillin plus nasal budesonide plus sea water nasal spray vs same PT | 1 (60,60); 12wks; [Guo L 2015] | -1.91 [-2.74, -1.08]* | -8.05 [-9.09, -7.01]* | -6.27 [-7.34, -5.20]* |

* significant difference

Abbreviations: C: control group; CHM: Chinese herbal medicine; CI: confidence interval; CRS: chronic rhinosinusitis; d: days; EoT: end of treatment; FU: follow-up; *I*^2^: index of heterogeneity; IM: integrative medicine; MD: mean difference; LK: Lund-Kennedy Endoscopic score; N: number; PT: pharmacotherapies; T: treatment group; vs: versus; wks: weeks.

Note: 1) baseline scores showed no significant difference between groups for each study in this comparison.

**S12 Table. MTT Meta-analysis results for CRS at end of treatment and change within treatment and control groups**

| **Comparison** | **N. studies (N. participants at EoT: T, C) Duration [study name]** | **T vs C at EoT; MD [95% CI] *I^2^*** | **T group change (baseline vs EoT); MD [95% CI] *I^2^*** | **C group change (baseline vs EoT); MD [95% CI] *I*^2^** |
| --- | --- | --- | --- | --- |
| **Nasal** **CHM vs inactive control^1^** | | | | |
| Steam inhalation of CHM decoction vs placebo inhalation | 1 (26,26); 3 days; [Yang L 2010] no BI | -315.34 [-508.07, -122.61]* sec | -436.61 [-622.00, -251.22]* sec | -29.39 [-261.34, 202.56] sec |
| **Nasal CHM vs nasal saline^1^** | | | | |
| Nasal YJPDHJ vs nasal 0.9% saline | 1 (30,31) 2wks [Wang J 2020] | -4.06 [-8.77, 0.65]* min | -8.64 [-13.19, -4.09]* min | -3.37 [-8.15, 1.41] min |
| **Oral CHM vs pharmacotherapy (PT) ^1^** | | | | |
| Oral BYHJ vs erythromycin enteric-coated capsules | 1 (30, 30, 25), 4 wks [Qiang JH 2011] no BI | -1.44 [-3.20, 0.32] min | -8.76 [-11.31, -6.21]* min | -4.91 [-7.38, -2.44]* min |
| Oral BYP vs erythromycin enteric-coated capsule |  | -0.06 [-1.88, 1.76] min | -5.47 [-7.53, -3.41]* min | As above |
| **Oral CHM plus pharmacotherapy vs the same PT (IM)^1^** | | | | |
| Oral BYTQKL plus PT vs PT: triamcinolone acetonide nasal spray | 1 (63,63); 10d; [Chu XY 2017] | -264.11 [-398.08, -130.14]* sec | -414.37 [-553.41, -275.33]* sec | -207.75 [-349.30, -66.20]* sec |
| Oral BYTQKL plus PT vs PT: triamcinolone acetonide nasal spray | 1 (74,74); 30d; [Deng QH 2016] | -199.71 [-307.08, -92.34]* sec | -403.65 [-515.96, -291.34]* sec | -150.87 [-278.74, -23.00]* sec |
| **Pool**: Oral BYTQKL plus PT vs PT: triamcinolone acetonide nasal spray | 2 (137,137); 10d-30d; [Chu XY 2017, Deng QH 2016] | -224.90 [-308.68, -141.11]* sec 0% | -407.88 [-495.25, -320.52]* sec 0% | -176.43 [-271.32, -81.54]* sec 0% |

* significant difference

Abbreviations: C: control group; CHM: Chinese herbal medicine; CI: confidence interval; CRS: chronic rhinosinusitis; d: days; EoT: end of treatment; *I*^2^: index of heterogeneity; IM: integrative medicine; MD: mean difference; min: minute; MTT: mucociliary transport time; N: number; PT: pharmacotherapies; sec: second; T: treatment group; vs: versus.

Note: 1) baseline scores showed no significant difference between groups for each study in this comparison.

**S13 Table. MTR Meta-analysis results for CRS at end of treatment and change within treatment and control groups**

| **Comparison** | **N. studies (N. participants at EoT: T, C) Duration [study name]** | **T vs C at EoT; MD [95% CI] *I^2^*** | **T group change (baseline vs EoT); MD [95% CI] *I^2^*** | **C group change (baseline vs EoT); MD [95% CI] *I*^2^** |
| --- | --- | --- | --- | --- |
| **Oral CHM vs pharmacotherapy (PT)^1^** | | | | |
| Oral BYGBF vs cefadroxil^2^ | 1 (27, 28), 3 wks [ Wang H 2009] no BI | 1.11 [0.30, 1.92]* mm/min | 3.82 [2.97, 4.67]* mm/min | 2.80 [2.01, 3.59]* mm/min |
| **Oral CHM plus pharmacotherapy vs the same PT (IM)^1^** | | | | |
| Oral BYKT plus PT vs PT: clarithromycin plus gentamicin sulfate injection + dexamethasone sodium phosphate injection + normal saline nasal wash | 1 (60,60); 8wks [Liao WT 2020] | 1.43 [0.96, 1.90]* mm/min | 2.82 [2.38, 3.26]* mm/min | 1.37 [0.96, 1.78]* mm/min |

* significant difference

Abbreviations: C: control group; CHM: Chinese herbal medicine; CI: confidence interval; CRS: chronic rhinosinusitis; d: days; EoT: end of treatment; *I*^2^: index of heterogeneity; IM: integrative medicine; MD: mean difference; min: minute; mm: millimetre; MTR: mucociliary transport rate; N: number; PT: pharmacotherapies; T: treatment group; vs: versus.

Notes: 1) baseline scores showed no significant difference between groups for each study in this comparison; 2. both groups received a nasal spray of 0.25% chloramphenicol plus 5 mg dexamethasone.

**S14 Table.** **SF-36 Meta-analysis results for CRS at end of treatment and change within treatment groups and control groups**

| **Outcome measure** | **Comparison** | **N. studies (N. participants at EoT: T, C) Duration [study name]** | **T vs C at EoT; MD [95% CI] *I^2^*** | **T group change (baseline vs EoT); MD [95% CI] *I^2^*** | **C group change (baseline vs EoT); MD [95% CI] *I*^2^** |
| --- | --- | --- | --- | --- | --- |
| **SF36® total scores** | Oral LDXGT vs placebo**^1,2^** | 1 (30,30) 2wks [Zhou L 2013] | 6.50 [-25.74, 38.74] | -16.20 [-51.33, 18.93] | -57.80 [-89.42, -26.18]* |
| **MOS SF-36® subscales** | **CHM plus pharmacotherapy vs the same pharmacotherapy (PT)** | | | | |
| vitality | Oral LDXGT plus oral clarithromycin plus mometasone furoate nasal spray vs same PT**^3,4^** | 1 (30,30) 12wks [Chen TT 2017] | 1.83 [-3.45, 7.11] | 8.33 [3.39, 13.27]* | 8.84 [3.31, 14.37]* |
| physical functioning |  |  | -0.50 [-3.37, 2.37] | 3.65 [-2.29, 9.59] | 3.33 [-2.88, 9.54] |
| bodily pain |  |  | 7.37 [1.44, 13.30]* | 11.63 [4.05, 19.21]* | 6.76 [-1.40, 14.92] |
| general health perceptions |  |  | 7.33 [3.35, 11.31]* | 19.16 [13.46, 24.86]* | 12.17 [7.04, 17.30]* |
| physical role functioning |  |  | 5.84 [-2.51, 14.19] | 18.34 [7.35, 29.33]* | 10.83 [-2.01, 23.67] |
| emotional role functioning |  |  | 13.34 [1.34, 25.34]* | 27.78 [13.56, 42.00]* | 18.89 [3.58, 34.20]* |
| social role functioning |  |  | 7.04 [1.31, 12.77]* | 25.56 [18.49, 32.63]* | 17.78 [8.70, 26.86]* |
| mental health |  |  | - 1. -3.63, 5.23] | 4.00 [-1.37, 9.37] | 0.80 [-4.66, 6.26] |

* significant difference

Abbreviations: C: control group; CHM: Chinese herbal medicine; CI: confidence interval; CRS: chronic rhinosinusitis; EoT: end of treatment; *I*^2^: index of heterogeneity; MD: mean difference; MOS: Medical Outcomes Study; SF-36: 36-Item Short Form Survey; N: number; PT: pharmocotherapies; T: test group; vs: versus.

Notes: 1) lower score indicates improvement; 2) at baseline the test group of this study (Zhou L 2013) had a lower SF-36 total score than the placebo group (MD -35.10 [-69.66, -0.54]); 3) higher score indicates improvement; 4) baseline scores showed no significant difference between groups for each item in this comparison.

**S15 Table. Details of reported adverse events from included studies**

| **Study name; duration; participants (T, C)** | **Test group (T)** | **Control group (C)** | **Adverse events** |
| --- | --- | --- | --- |
| **Chronic rhinosinusitis (CRS)** | | | |
| Du JW 2016;20d; (44,45) | Oral BDYKFY plus clarithromycin | clarithromycin | T:3 (diarrhea=1, skin rash=1, nausea=1); C:2 (nausea=1, elevated transaminase=1). |
| Wang C 2014; 12wks; (48,48) | Oral BYSKFY plus clarithromycin plus eucalyptol, limonene and pinene capsules | clarithromycin plus eucalyptol, limonene and pinene capsules | T:3 (gastrointestinal discomfort=3); C:2 (gastrointestinal discomfort=2). |
| Wang KQ 2016; 12wks; (84,43) | Nasal BYT plus cefaclor plus budesonide | cefaclor plus budesonide | T:2 (mild nose bleeding=1, headache=1); C:0. |
| **Pooled result CRS, 3 studies (176,136)** | **CHM plus PT, 8 AEs (4.5%)** | **PT, 4 AEs (2.9%)** | **RR 1.64 [0.52, 5.12] 0%** |
| Zhou L 2013; 2wks; (34,28) | Oral LDXGT | placebo | T:1 (diarrhea=1); C:0. |
| Lin L 2020; 30d; (70,70) | Oral LHQWKL | placebo | T:8 (mild stomachache and diarrhea); C:3 (mild stomachache and diarrhea) |
| **Pooled result CRS, 5 studies (280,234)** | **17 AEs (6.0%)** | **7 AEs (3.0%)** | **RR** **2.06 [0.90, 4.69] I^2^ 0%** |
| Yang L 2010, Jiang RS 2012; Liu JB 2011; Hu FL 2015; Liu Q 2015; Zhang YF 2015; Zhu XP 2017, Wang H 2009; Cai CJ 2019 | 9 studies of various CHMs | Various PTs | T: 0; C: 0 |
| **Total result CRS, 14 studies (613,564)** | **17 AEs (2.8%)** | **7 AEs (1.2%)** | **RR 2.23 [0.93, 5.35]** including 9 studies with 0 AEs |
| Chen TT 2017; Chu XY 2017; Dai RZ 2015; Deng QH 2016; Guo L 2015; Hong HY 2015; Liu HL 2017; Wang G 2013; Wang P 2015; Xiong J 2016; Zhang LY 2015; Zhang XQ 2015; Liao WT 2020; Wang J 2020; Qiang JH 2011; Wu MM 2022 | 16 studies of various CHMs | Various PTs | AEs were not reported |
| **Acute rhinosinusitis (ARS)** | | | |
| Huang JY 2017; 10 days (95,95) | Oral BYKT plus oral cefuroxime tablets | oral cefuroxime tablets | T: 2 (allergic reactions), 3 (elevated liver enzymes); C: 2 (allergic reactions), 2 (elevated liver enzymes) |
| **Pooled result ARS** | **5 AEs** | **4 AEs** | **RR 1.25 [0.35, 4.51]** |
| Li MJ 2014; Lin L 2015; Zhong MR 2020 | 3 studies of various CHMs | Various PTs | AEs were not reported |

Abbreviations: ALT: Alanine aminotransferase; AST: Aspartate aminotransferase; C: control group; d: days; PT: pharmacotherapy; T: test group; wks: weeks.
