# Supplementary material for: Chinese herbal therapy in the management of rhinosinusitis—A systematic review and meta-analysis
Source: PLoS One. 2022 Dec 1;17(12):e0278492. doi: 10.1371/journal.pone.0278492 (PMC9714754; doi:10.1371/journal.pone.0278492)
Supplement: Attachment — Submitted filename: Rebuttal leter PLOS ONE 22 08 2022.docx [file pone.0278492.s003.docx]

**Rebuttal letter**

Dear Editors

We wish to respond to the comments received from the Staff Editor regarding our previous submission PONE-D-22-21862, which were as follows:

PONE-D-22-21862
Can Chinese herbal therapy improve outcomes for rhinosinusitis? - A systematic review and meta-analysis
PLOS ONE

Dear Dr. Xue,

Thank you for submitting your manuscript to PLOS ONE. After careful consideration, we have decided that your manuscript does not meet our criteria for publication and must therefore be rejected.

While PLOS ONE does consider systematic reviews and meta-analyses for publication, we require that these submissions address a clear and defined research question, conduct a systematic and comprehensive literature review; and use clearly reported, reproducible, and systematic methods to identify, select, and critically appraise relevant research. Our author guidelines for systematic reviews are at [https://journals.plos.org/plosone/s/submission-guidelines#loc-systematic-reviews-and-meta-analyses](https://aus01.safelinks.protection.outlook.com/?url=https%3A%2F%2Fjournals.plos.org%2Fplosone%2Fs%2Fsubmission-guidelines%23loc-systematic-reviews-and-meta-analyses&data=05%7C01%7Cbrian.may%40rmit.edu.au%7C8ef26b8603f747d5fe3a08da78012960%7Cd1323671cdbe4417b4d4bdb24b51316b%7C0%7C0%7C637954243645502013%7CUnknown%7CTWFpbGZsb3d8eyJWIjoiMC4wLjAwMDAiLCJQIjoiV2luMzIiLCJBTiI6Ik1haWwiLCJXVCI6Mn0%3D%7C3000%7C%7C%7C&sdata=992KQcZCy3cOfU4%2FIsopo7tUdBXAKfgOC3DmNjEscKU%3D&reserved=0).

In the case of your submission, we are concerned that the literature review was completed in June 2021. Systematic and scoping reviews should provide an up-to-date overview of the research topic covered. Since the literature search was completed over a year ago, we are concerned that this study does not meet this requirement.

As a result of these concerns, we cannot consider the manuscript for publication at PLOS ONE at this time. However, if you are able to address the concerns discussed above, we may be able to consider a revised version of the manuscript. This would however require a substantial amount of work and thus, would be considered as a new submission. If you are able to revise the manuscript to respond to the concerns noted above, please refer to the original submission in the covering letter and accompany the submission by a rebuttal letter that responds to the comments made on the original manuscript.

I am sorry that we cannot be more positive on this occasion, but hope that you appreciate the reasons for this decision.

Kind regards,

Lucinda Shen, MSc
Staff Editor
PLOS ONE

Additional Editor Comments (if provided):

[Note: HTML markup is below. Please do not edit.]

Reviewers' comments:

[NOTE: If reviewer comments were submitted as an attachment file, they will be attached to this email and accessible via the submission site. Please log into your account, locate the manuscript record, and check for the action link "View Attachments". If this link does not appear, there are no attachment files.]

**Our responses and actions**

We thank the Staff Editor for the comments. The principal concern was that the literature search was completed over a year ago, so the systematic review was not up-to-date.

Therefore, we conducted additional searches of the English and Chinese language databases and clinical trial registries for the years 2021 and 2022, so the current search includes all results up until the first week of August 2022. This search resulting in one addition RCT being included. It also found no recent systematic reviews on this topic, although there was a systematic review protocol for a related topic published in PLOS ONE.

The manuscript, tables and meta-analyses have all been updated accordingly. Consequently, the revised paper is up to date.

We also revised the paper thoroughly to improve the writing style and clarity.

We request that PLOS ONE consider this paper for peer review.

Yours sincerely

Distinguished Professor Charlie C Xue

RMIT University
